# Efficacy of dietary supplements as an adjunctive therapy for polycystic ovary syndrome: an umbrella meta-analysis

**DOI:** 10.3389/fnut.2025.1705284

**Published:** 2025-10-29

**Authors:** Rutong Wang, Kongwei Huang, Mengyao Wang, Wenhui Zou, Yujun Huang, Wei Jiang, Yingqi Feng, Huilong Shen, Xiaocan Lei

**Affiliations:** ^1^Clinical Anatomy & Reproductive Medicine Application Institute, Hengyang Medical School, University of South China, Hengyang, Hunan, China; ^2^Hengyang Hospital of Hunan Normal University & Hengyang Central Hospital, Hengyang, Hunan, China

**Keywords:** dietary supplements, polycystic ovary syndrome, umbrella meta-analysis, bibliometrics, future trend

## Abstract

**Introduction:**

Polycystic ovary syndrome (PCOS) affects 5–15% of reproductive-aged women and involves significant metabolic dysregulation, for which nutritional interventions show therapeutic potential. Methods: This umbrella meta-analysis synthesizes evidence from 46 randomized trials (*n* = 30,133) to evaluate dietary supplements targeting core PCOS pathways.

**Methods:**

This umbrella meta-analysis synthesizes evidence from 46 randomized trials (*n* = 30,133) to evaluate dietary supplements targeting core PCOS pathways.

**Results:**

Key nutraceuticals demonstrate clinically relevant benefits: myo-inositol significantly improves insulin sensitivity (HOMA-IR SMD = −0.81) and SHBG levels (SMD = 9.65) by enhancing glucose transporter activity; probiotics reduce systemic inflammation (CRP SMD = −0.82) via gut-microbiota modulation; omega-3 fatty acids ameliorate dyslipidemia (LDL-C SMD = −9.57; HDL-C SMD = 2.31) through anti-inflammatory mechanisms. Plant-derived compounds like curcumin lower fasting glucose (SMD = −3.43) via NF-ĸB pathway inhibition, while green tea catechins reduce adiposity. Significant heterogeneity arises from variations in supplement bioavailability, dosing protocols, and patient metabolic phenotypes. Nevertheless, consistent evidence confirms that targeted nutrient supplementation modulates insulin signaling, lipid metabolism, and hormonal balance in PCOS. Emerging research priorities include personalized nutrition protocols leveraging nutrigenomic interactions and antioxidant-rich formulations (e.g., vitamin E, selenium).

**Discussion:**

This work establishes a mechanistic foundation for integrating evidence-based nutraceuticals—particularly myo-inositol, probiotics, and omega-3s—into PCOS management, offering clinically actionable strategies while highlighting needs for standardized dosing and bioavailability studies.

**Systematic review registration:**

https://www.crd.york.ac.uk/PROSPERO/view/CRD42024602681.

## 1 Introduction

Over the past half-century, global birth rates have plummeted significantly (1). Polycystic ovary syndrome (PCOS) is a leading cause of female infertility, affecting 5–15% of women of reproductive age (2, 3). The syndrome is characterized by pathological features such as abnormal secretion of gonadotropins, ovarian folliculogenesis, steroidogenesis, insulin secretion, and adipose tissue function (4). Studies have indicated that PCOS is often associated with symptoms like central obesity and insulin resistance (5). Weight gain and obesity exacerbate insulin resistance and metabolic syndrome features (6). In individuals with PCOS, excess weight contributes to reproductive and metabolic risks (7–9). Weight gain and obesity in women with PCOS can also lead to worsened insulin resistance and metabolic dysfunction (mediated by further impairment of the PI3 kinase downstream insulin receptor pathway), as well as the typical reproductive disorders and hyperandrogenism associated with the condition (10). An umbrella meta-analysis suggests that insulin resistance is considered a primary factor in the pathophysiology of PCOS and is involved in the development of hyperandrogenemia and reproductive dysfunction through various mechanisms (11). Therefore, nutrition and dietary interventions play a crucial role in the progression or management of the disease (12).

Previous research has shown metabolic disturbances in many nutrients in PCOS patients, such as vitamin D, minerals, and the ratio of omega-6 to omega-3 fatty acids (13). Supplementation with natural molecules like inositol, vitamin E, vitamin D, and omega-3 may help alleviate pathological symptoms of PCOS, such as oocyte immaturity, insulin resistance, hyperandrogenism, oxidative stress, and inflammation (14). Alesi et al. (15) highlighted the potential benefits of specific nutrients—including vitamins (e.g., B-12, inositol, vitamin D, E, K), minerals (e.g., calcium, selenium), and other compounds (e.g., omega-3 fatty acids, probiotics)—for PCOS management. A network umbrella meta-analysis by Hu et al. (16) shows that carnitine, inositol, and probiotics are effective in reducing weight and body mass index (BMI). Omega-3 is effective in lowering fasting blood glucose (FBG), chromium is effective in lowering fasting insulin (FINS), and coenzyme Q10 is the most effective in improving lipid metabolism (16). However, a systematic review by Menichini et al. (17) notes that while some nutritional supplements (such as inositol, probiotics, vitamin D, and E) show potential benefits in certain aspects, these results are not consistent and further research is needed to confirm their efficacy. A review article published in *J Turk Ger Gynecol Assoc* points out that although some studies suggest that nutritional supplements may be beneficial for the metabolic and endocrine functions of PCOS (18). Several systematic reviews and umbrella meta-analyses have indicated that while individual studies show potential benefits of certain nutritional supplements, the overall evidence is insufficient and more high-quality studies are needed to validate these findings (19).

The current umbrella meta-analysis aims to critically evaluate the efficacy of nutritional supplements in the treatment of PCOS, intending to provide a more nuanced understanding of their potential benefits and to address the existing discrepancies in the literature.

## 2 Materials and methods

This study adheres to the Preferred Reporting Items for Systematic Reviews and Meta-Analyses (PRISMA) statement (20). The protocol for this systematic review and umbrella meta-analysis has been prospectively registered in the PROSPERO database (registration number: CRD42024600102).

### 2.1 Eligibility criteria for this review

This review includes umbrella meta-analyses evaluating the efficacy of nutritional supplements—such as n-3 polyunsaturated fatty acids, alpha-lipoic acid, polyphenols, minerals, melatonin, vitamins, inositol, probiotics, and L-carnitine—compared to placebo or alternative treatments in women diagnosed with PCOS using the Rotterdam criteria (21).

### 2.2 Search strategy

We conducted a comprehensive literature search across PubMed, Web of Science, Embase, and Scopus up to September 13, 2024, identifying umbrella meta-analyses on nutritional supplement interventions for PCOS, and included a thorough review of relevant publications, review articles, and the reference lists of included studies. To further expand our literature resources, We manually reviewed reference lists of included studies and contacted field experts to identify additional or unpublished sources. Our search strategy skillfully combined MeSH terms and keywords, and our search was limited to human studies without language restrictions. The detailed search strategy, keywords, and specific search syntax for each database are listed in Appendix A. We applied specific exclusion criteria to ensure the quality and relevance of the included studies.

Exclusion Criteria:

Studies were excluded if they:

- Were non-randomized controlled trials (non-RCTs)
- Included fewer than 20 participants
- Were not published in peer-reviewed journals
- Did not focus on nutritional supplement treatment for PCOS
- Lacked sufficient data for analysis

By applying these exclusion criteria, we aimed to include only high-quality studies that directly addressed the research question.

### 2.3 Data synthesis and quality assessment

Two reviewers (RTW and MYW) independently screened titles and abstracts using predefined criteria. Full texts of potentially eligible studies were assessed, with disagreements resolved by consensus or a third reviewer (XCL). Data extracted from the included studies encompassed publication year, sample size, geographical location of the study, duration of nutritional supplementation, standardized mean difference (SMD), mean difference (MD), odds ratio (OR), relative risk (RR), and their corresponding 95% confidence intervals (CI). To standardize effect sizes, odds ratios (ORs) were converted to relative risks (RRs), and mean differences (MDs) to standardized mean differences (SMDs).

### 2.4 Statistical analysis

Statistical analyses were conducted using STATA version 16.0 (StataCorp, College Station, TX, United States). A random-effects model was applied when I^2^ exceeded 50%, accounting for heterogeneity across studies. Sensitivity analyses assessed the robustness of findings, with all estimates reported as 95% confidence intervals (CI).

## 3 Results

### 3.1 Study selection and characteristics

**Figure 1:**
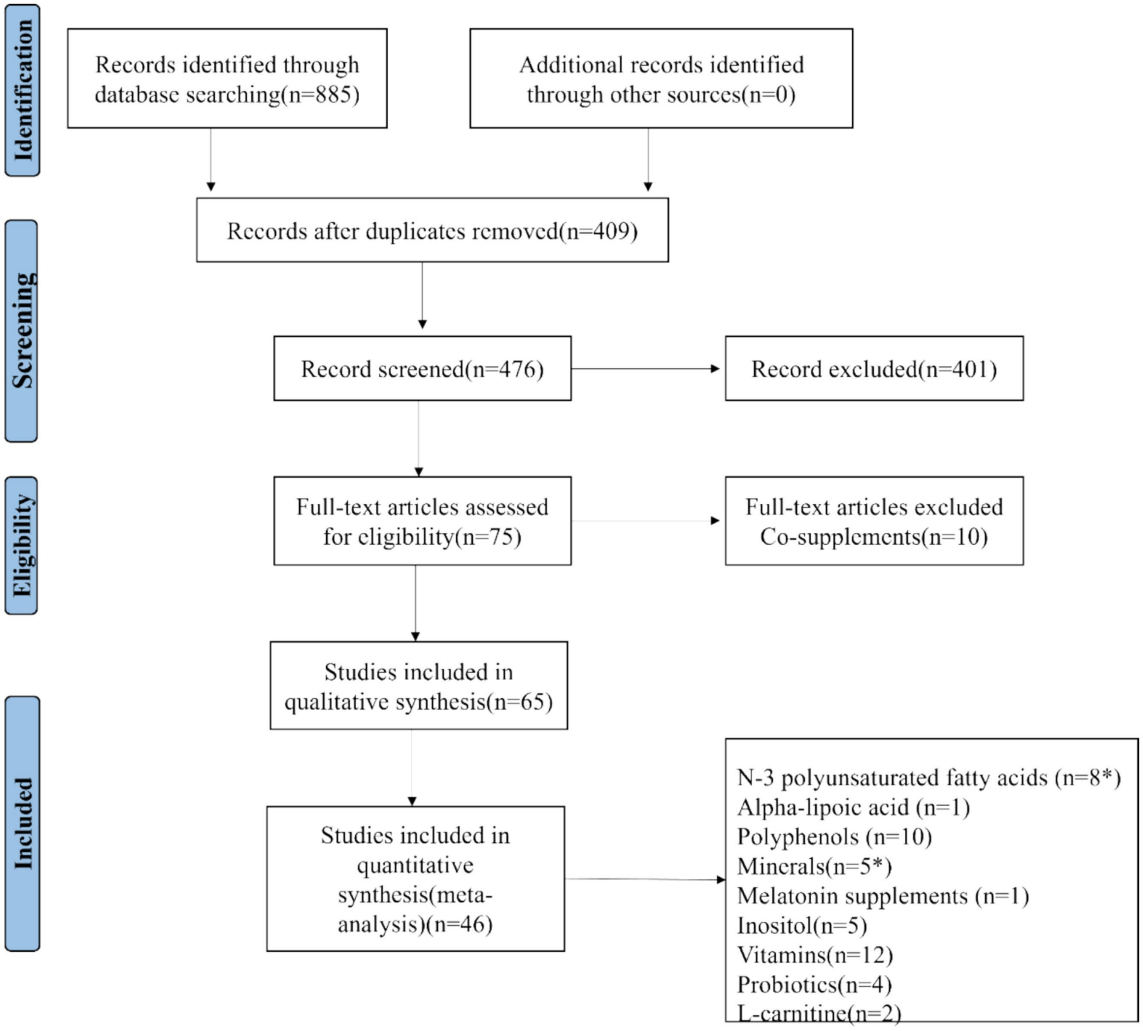
The process study selection shown on PRISMA flow chart.

The initial search yielded 885 umbrella meta-analysis articles on the treatment of PCOS with nutritional supplements: 130 from PubMed, 227 from Embase, 248 from Scopus, and 280 from Web of Science. No unpublished studies were identified. Among these, 409 studies were determined to be duplicates. After rigorous screening of the titles and abstracts of the remaining 476 articles, 75 articles were assessed in full text, resulting in 65 articles that met the qualitative synthesis criteria. For quantitative synthesis, a total of 46 studies were ultimately included (22–62). The process is depicted in Figure 1. The selected 46 studies comprised 30,133 female participants. The studies that met the inclusion criteria spanned from 2016 to 2024. These investigations were geographically distributed across 12 countries: Australia (1 study), Iran (7 studies), Brazil (2 studies), China (11 studies), Saudi Arabia (3 studies), United States (1 study), Taiwan (1 study), Mexico (1 study), Hungary (1 study), United Kingdom (1 study), Greece (1 study), and Malaysia (1 study). Each randomized controlled trial included in the umbrella meta-analysis utilized nutritional supplements as a therapy for PCOS. The detailed characteristics of the included umbrella meta-analyses are summarized in Appendix C: Characteristics of umbrella meta-analyses examining the effects of nutritional supplements on PCOS.

### 3.2 Umbrella meta-analysis results

#### 3.2.1 N-3 unsaturated fatty acids

**Figure 2:**
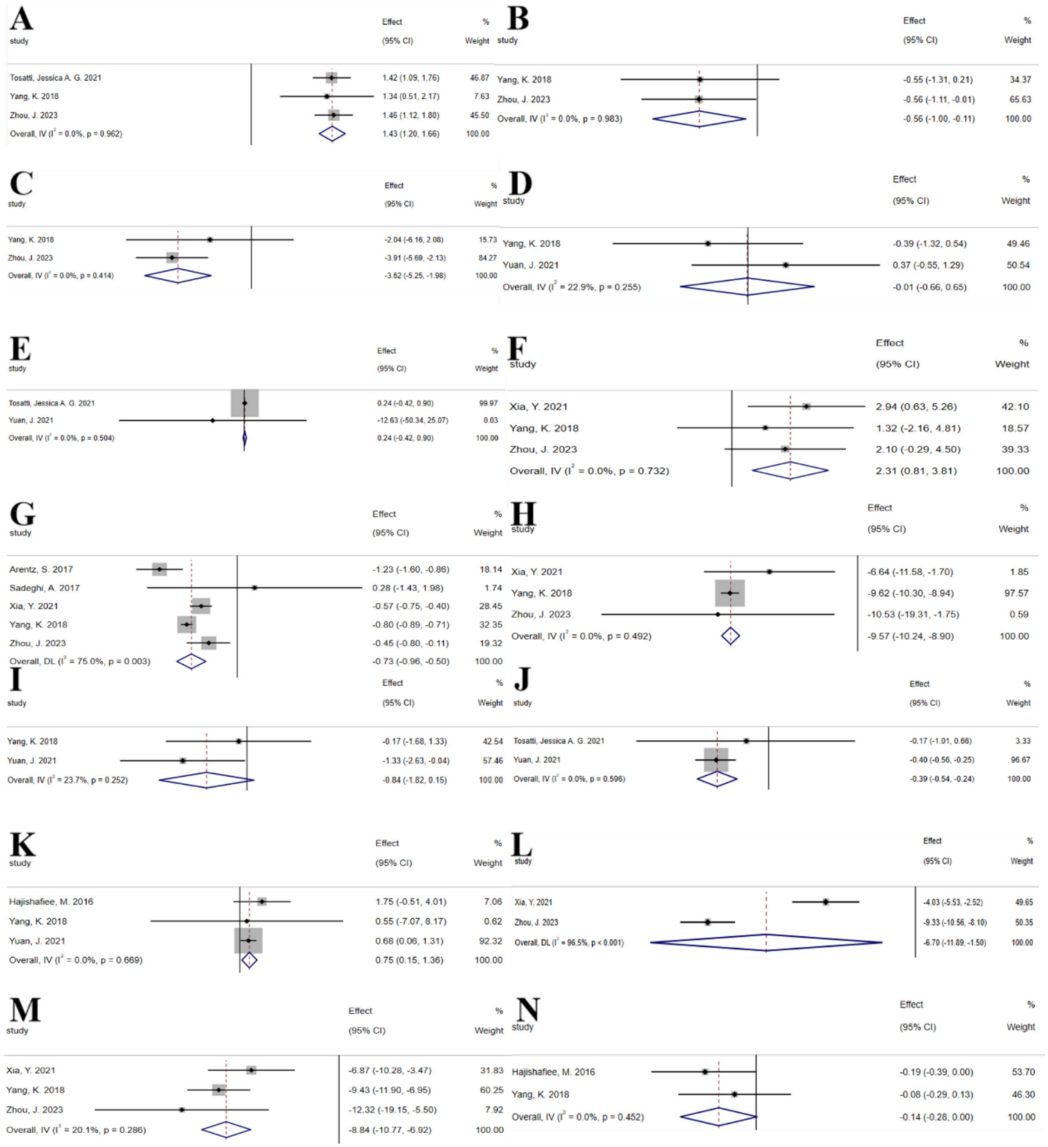
N-3 unsaturated fatty acids forest plot. **(A)** Adiponectin concentration, **(B)** BMI, **(C)** Fasting glucose, **(D)** FSH, **(E)** GSH, **(F)** HDL-c, **(G)** HOMA-IR, **(H)** LDL-c, **(I)** LH, **(J)** MDA, **(K)** SHBG, **(L)** TG, **(M)** Total cholesterol, **(N)** Total testosterone.

The umbrella meta-analysis encompassed 8 studies reporting on n-3 unsaturated fatty acids (22–29). A total of 29 relevant outcomes were recorded, with the experimental group receiving oral n-3 unsaturated fatty acids and the control group receiving a placebo. Notably, adiponectin levels (SMD: 1.43; 95% CI: 1.20, 1.66; *I*^2^ = 0.0%, *p* = 0.962), BMI (SMD: −0.56; 95% CI: −1.00, −0.11; *I*^2^ = 0.0%, *p* = 0.983), fasting blood glucose (SMD: −3.62; 95% CI: −5.25, −1.98; *I*^2^ = 0.0%, *p* = 0.414), FSH (SMD: −0.01; 95% CI: −0.66, 0.65; *I*^2^ = 22.9%, *p* = 0.255), GSH (SMD: 0.24; 95% CI: −0.42, 0.90; *I*^2^ = 0.0%, *p* = 0.504), HDL-c (SMD: 2.31; 95% CI: 0.81, 3.81; *I*^2^ = 0.0%, *p* = 0.732), HOMA-IR (SMD: −0.73; 95% CI: −0.96, −0.50; *I*^2^ = 75.0%, *p* = 0.003), LDL-C (SMD: −9.57; 95% CI: −10.24, −8.90; *I*^2^ = 0.0%, *p* = 0.492), LH (SMD: −0.84; 95% CI: −1.82, 0.15; *I*^2^ = 23.7%, *p* = 0.252), MDA (SMD: −0.39; 95% CI: −0.54, −0.24; *I*^2^ = 0.0%, *p* = 0.596), SHBG (SMD: 0.75; 95% CI: 0.15, 1.36; *I*^2^ = 0.0%, *p* = 0.669), TG (SMD: −6.70; 95% CI: −11.89, −1.50; *I*^2^ = 96.5%, *p* < 0.001), total cholesterol (SMD: −8.84; 95% CI: −10.77, −6.92; *I*^2^ = 20.1%, *p* = 0.286), and total testosterone (SMD: −0.14; 95% CI: −0.28, 0.00; *I*^2^ = 0.0%, *p* = 0.452) were among the outcomes assessed (Figure 2).

#### 3.2.2 Inositol

**Figure 3:**
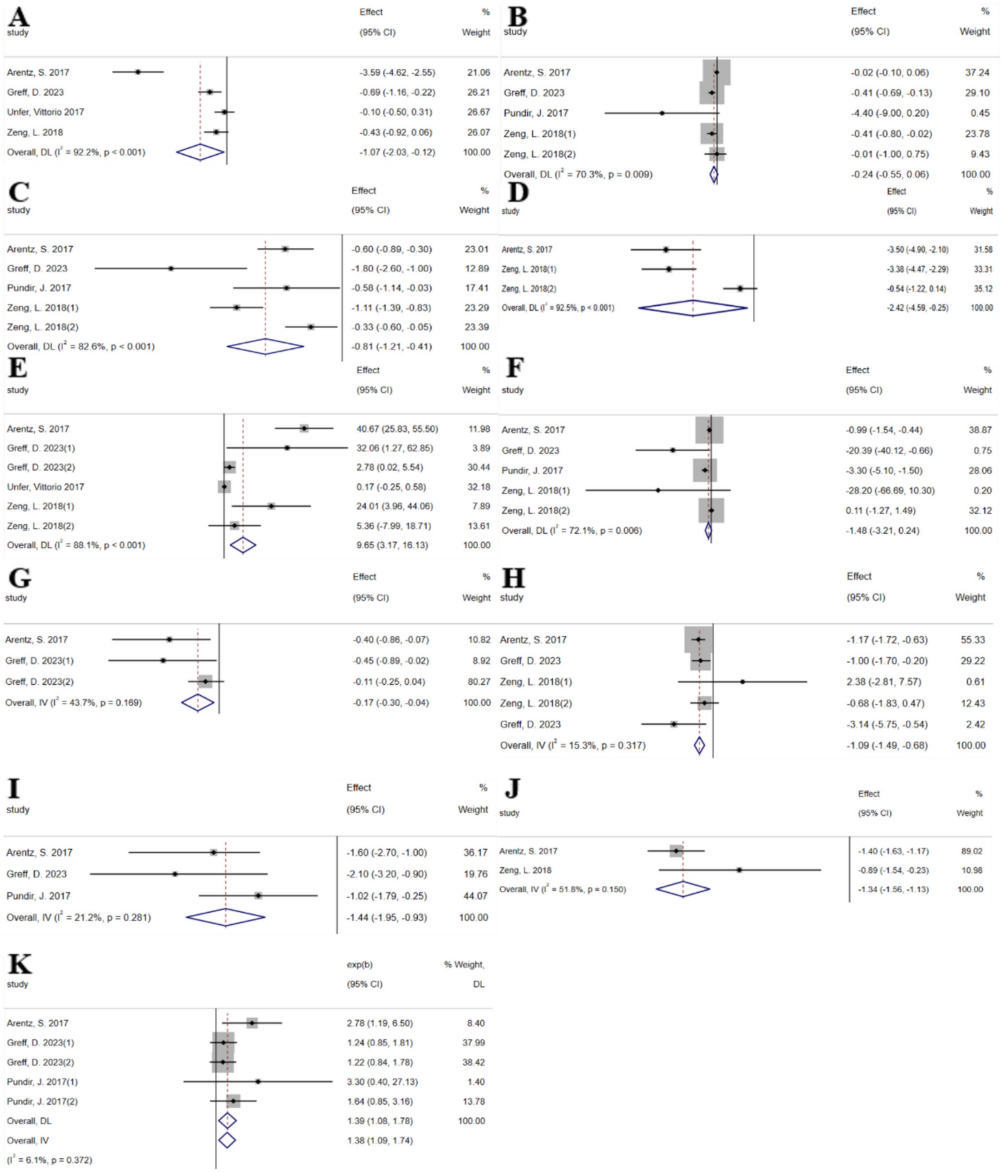
Inositol forest plot. **(A)** Androstenedione, **(B)** Free testosterone, **(C)** HOMA-IR, **(D)** LH, **(E)** SHBG, **(F)** Total testosterone, **(G)** BMI, **(H)** Fasting glucose, **(I)** Fasting insulin, **(J)** FSH, **(K)** Pregnancy.

The umbrella meta-analysis included 5 studies reporting on inositol (25, 30–33). A total of 24 relevant outcomes were recorded, with the experimental group receiving oral inositol and the control group receiving a placebo, except for the study by Zeng et al. (30), which used metformin as the control. Notably, androstenedione (SMD: −1.07; 95% CI: −2.03, −0.12; *I*^2^ = 92.2%, *p* < 0.001), free testosterone (SMD: −0.24; 95% CI: −0.55, 0.06; *I*^2^ = 70.3%, *p* = 0.009), HOMA-IR (SMD: −0.81; 95% CI: −1.21, −0.41; *I*^2^ = 82.6%, *p* < 0.001), LH (SMD: −2.42; 95% CI: −4.59, −0.25; *I*^2^ = 92.5%, *p* < 0.001), SHBG (SMD: 9.65; 95% CI: 3.17, 16.13; *I*^2^ = 88.1%, *p* < 0.001), total testosterone (SMD: −1.48; 95% CI: −3.21, 0.24; *I*^2^ = 72.1%, *p* = 0.006), BMI (SMD: −0.17; 95% CI: −0.30, −0.04; *I*^2^ = 43.7%, *p* = 0.169), fasting blood glucose (SMD: −1.09; 95% CI: −1.49, −0.68; *I*^2^ = 15.3%, *p* = 0.317), fasting insulin (SMD: −1.44; 95% CI: −1.95, −0.93; *I*^2^ = 21.2%, *p* = 0.281), FSH (SMD: −1.34; 95% CI: −1.56, −1.13; *I*^2^ = 51.8%, *p* = 0.150), and pregnancy rate (RR: 1.38; 95% CI: 1.09, 1.74; *I*^2^ = 6.1%, *p* = 0.372) were among the outcomes assessed (Figure 3). Given the significant high heterogeneity observed in the related outcomes, sensitivity analyses were conducted (Supplementary Figure S1). After excluding studies with considerable heterogeneity, androstenedione (SMD: −1.47; 95% CI: −2.83, −0.12; *I*^2^ = 93.4%, *p* < 0.001), free testosterone (SMD: −0.39; 95% CI: −0.61, −0.17; *I*^2^ = 18.3%, *p* = 0.299), HOMA-IR (SMD: −0.90; 95% CI: −1.51, −0.30; *I*^2^ = 74.5%, *p* = 0.020), LH (SMD: −3.43; 95% CI: −4.29, −2.57; *I*^2^ = 0.0%, *p* = 0.895), and SHBG (SMD: 19.17; 95% CI: 2.79, 35.54; *I*^2^ = 87.1%, *p* < 0.001) were re-evaluated (Supplementary Figure S2).

#### 3.2.3 Plant polyphenols

##### 3.2.3.1 Curcumin

**Figure 4:**
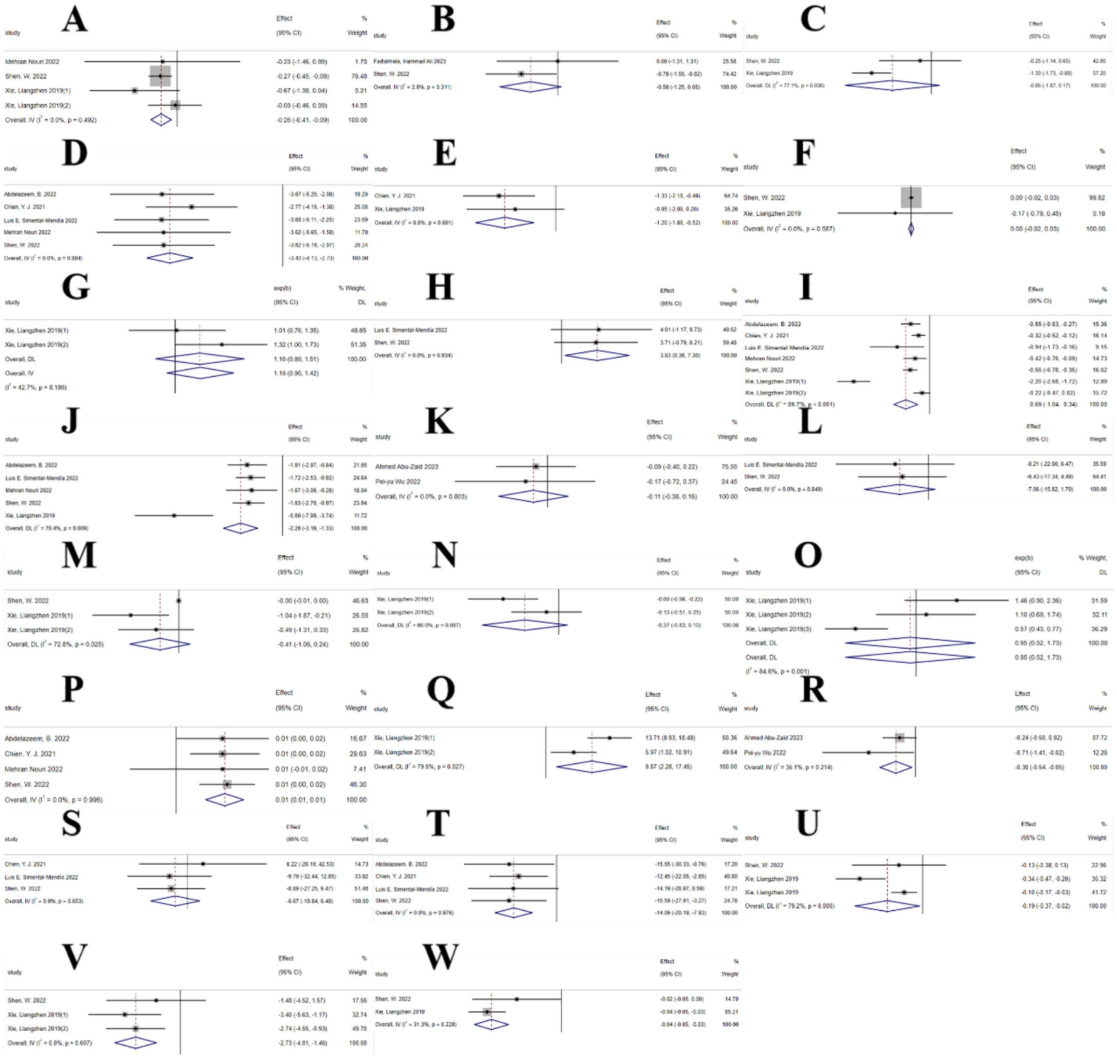
Curcumin forest plot. **(A)** BMI, **(B)** C-reactive protein, **(C)** FAI, **(D)** Fasting blood glucose, **(E)** Fasting insulin, **(F)** FSH, **(G)** Gastrointestinal adverse events, **(H)** HDL-c, **(I)** HOMA-IR, **(J)** Insulin, **(K)** LDL, **(L)** LDL-c, **(M)** LH, **(N)** Postprandial plasma glucose, **(O)** Pregnancy rates, **(P)** QUICKI, **(Q)** SHBG, **(R)** TC, **(S)** TG, **(T)** Total cholesterol, **(U)** Total testosterone, **(V)** Waist circumference, **(W)** WHR.

The umbrella meta-analysis included 7 studies reporting on curcumin (34–38). A total of 38 relevant outcomes were recorded, with the experimental group receiving oral curcumin and the control group receiving a placebo. Notably, BMI (SMD: −0.25; 95% CI: −0.41, −0.09; *I*^2^ = 0.0%, *p* = 0.492), C-reactive protein (SMD: −0.58; 95% CI: −1.25, 0.08; *I*^2^ = 2.6%, *p* = 0.311), FAI (SMD: −0.05; 95% CI: −1.87, 0.17; *I*^2^ = 77.1%, *p* = 0.036), fasting blood glucose (SMD: −3.43; 95% CI: −4.13, −2.73; I^2^ = 0.0%, *p* = 0.884), fasting insulin (SMD: −1.20; 95% CI: −1.88, −0.52; *I*^2^ = 0.0%, *p* = 0.601), FSH (SMD: 0.00; 95% CI: −0.02, 0.03; *I*^2^ = 0.0%, *p* = 0.587), gastrointestinal adverse events (RR: 1.16; 95% CI: 0.95, 1.42; *I*^2^ = 42.7%, *p* = 0.186), HDL-C (SMD: 3.83; 95% CI: 0.36, 7.30; *I*^2^ = 0.0%, *p* = 0.934), HOMA-IR (SMD: −0.69; 95% CI: −1.04, −0.34; *I*^2^ = 89.7%, *p* < 0.001), insulin (SMD: −2.26; 95% CI: −3.19, −1.33; *I*^2^ = 70.4%, *p* = 0.009), LDL (SMD: −0.11; 95% CI: −0.38, 0.16; *I*^2^ = 0.0%, *p* = 0.803), LDL-C (SMD: −7.06; 95% CI: −15.82, 1.70; *I*^2^ = 0.0%, *p* = 0.849), LH (SMD: −0.41; 95% CI: −1.06, 0.24; *I*^2^ = 72.8%, *p* = 0.025), postprandial blood glucose (SMD: −0.37; 95% CI: −0.83, 0.10; *I*^2^ = 66.0%, *p* = 0.087), pregnancy rate (RR: 0.95; 95% CI: 0.52, 1.73; *I*^2^ = 84.6%, *p* = 0.001), QUICK (SMD: 0.01; 95% CI: 0.01, 0.01; *I*^2^ = 0.0%, *p* = 0.996), SHBG (SMD: 9.87; 95% CI: 2.28, 17.45; *I*^2^ = 79.5%, *p* = 0.027), TC (SMD: −0.30; 95% CI: −0.54, −0.05; *I*^2^ = 35.1%, *p* = 0.214), TG (SMD: −6.67; 95% CI: −19.84, 6.49; *I*^2^ = 0.0%, *p* = 0.653), total cholesterol (SMD: −14.06; 95% CI: −20.19, −7.93; *I*^2^ = 0.0%, *p* = 0.976), total testosterone (SMD: −0.19; 95% CI: −0.37, −0.02; *I*^2^ = 79.2%, *p* = 0.008), waist circumference (SMD: −2.73; 95% CI: −4.01, −1.46; *I*^2^ = 0.0%, *p* = 0.607), and WHR (SMD: −0.04; 95% CI: −0.05, −0.03; *I*^2^ = 31.3%, *p* = 0.228) were among the outcomes assessed (Figure 4).

##### 3.2.3.2 Green tea

**Figure 5:**
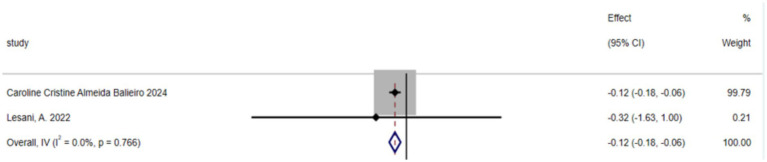
BMI forest plot of green tea.

The umbrella meta-analysis included 2 studies reporting on green tea (39). A total of 8 relevant outcomes were recorded, with the experimental group receiving oral green tea and the control group receiving a placebo. Notably, BMI (SMD: −0.12; 95% CI: −0.18, −0.06; *I*^2^ = 0.0%, *p* = 0.766) was among the outcomes assessed (Figure 5).

#### 3.2.4 Mineral substances

**Figure 6:**
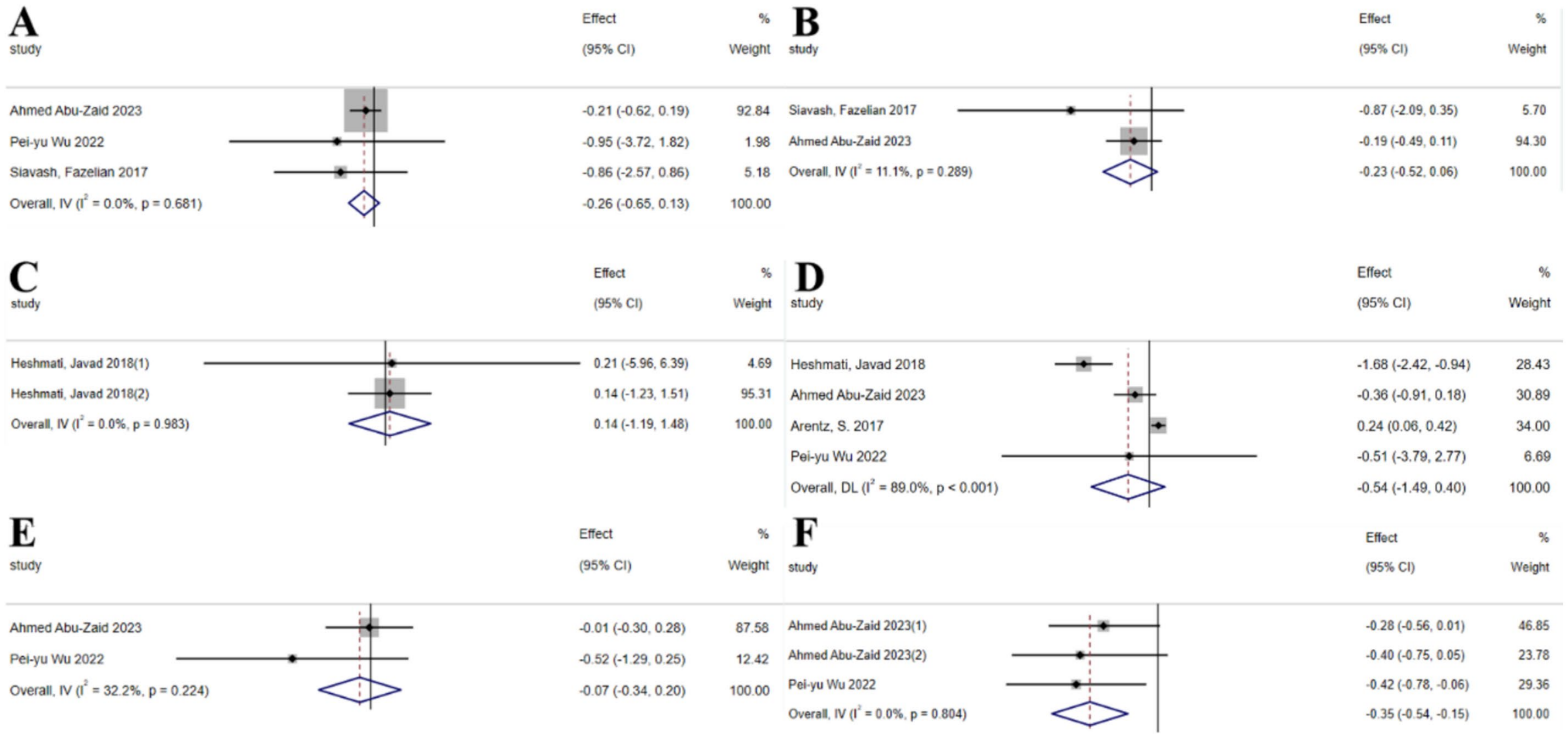
Mineral substance forest plot. **(A)** FBS, **(B)** FG, **(C)** FSI, **(D)** HOMA-IR, **(E)** SHBG, **(F)** Total testosterone.

The umbrella meta-analysis encompassed 5 studies reporting on mineral substances (25, 40–42), including three studies on selenium supplements (40, 41) and two studies on chromium supplements (42, 43). A total of 24 relevant outcomes were recorded, with the experimental group receiving oral chromium and selenium supplements and the control group receiving a placebo. Notably, FBS (SMD: −0.26; 95% CI: −0.65, 0.13; *I*^2^ = 0.0%, *p* = 0.681), FG (SMD: −0.23; 95% CI: −0.52, 0.06; *I*^2^ = 11.1%, *p* = 0.289), FSI (SMD: 0.14; 95% CI: −1.19, 1.48; *I*^2^ = 0.0%, *p* = 0.983), HOMA-IR (SMD: −0.54; 95% CI: −1.49, 0.40; *I*^2^ = 89.0%, *p* < 0.001), and SHBG (SMD: −0.07; 95% CI: −0.34, 0.20; *I*^2^ = 32.2%, *p* = 0.224), as well as total testosterone (SMD: −0.35; 95% CI: −0.54, −0.15; *I*^2^ = 0.0%, *p* = 0.804) were among the outcomes assessed (Figure 6).

#### 3.2.5 Vitamins

##### 3.2.5.1 Vitamin D

**Figure 7:**
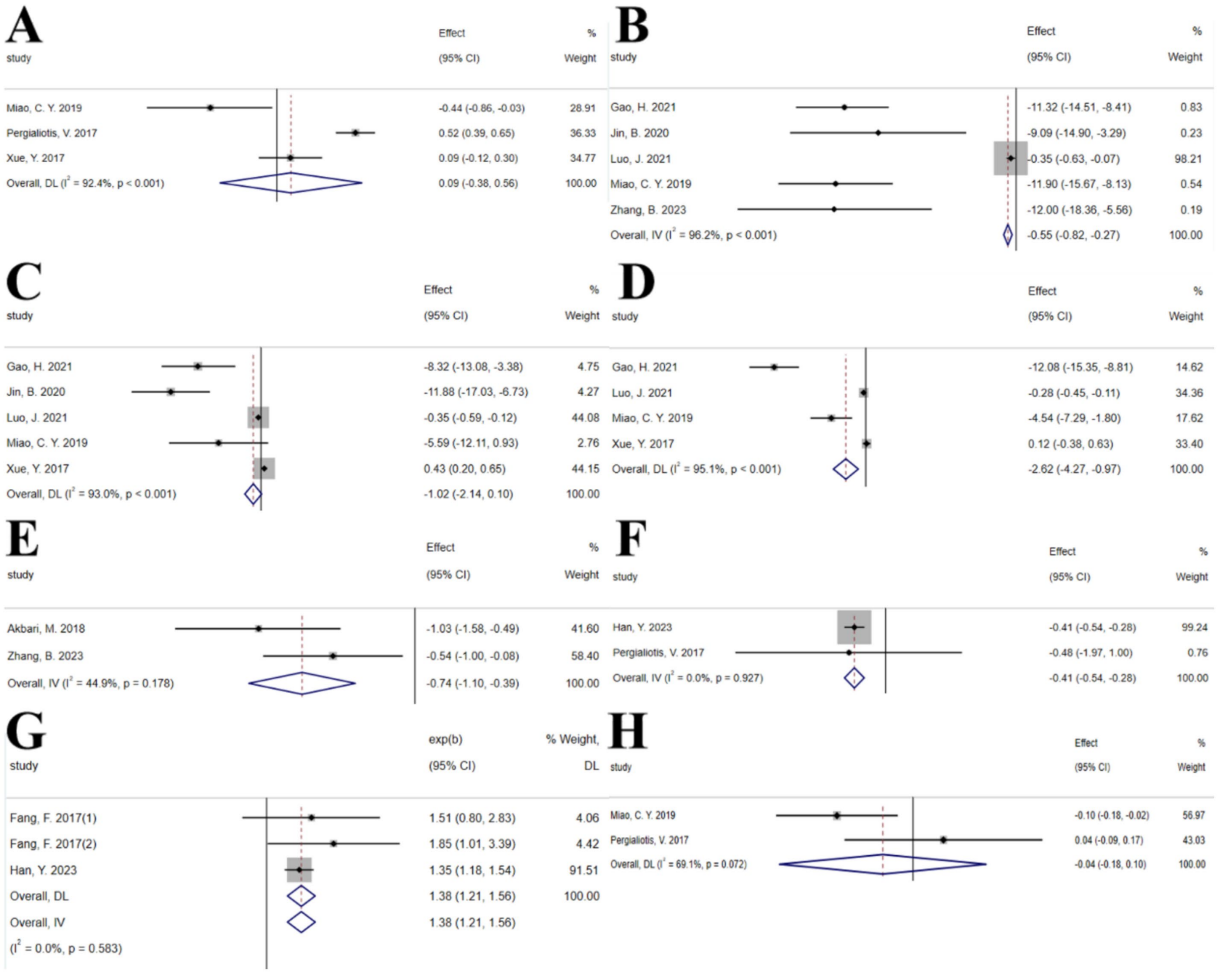
Vitamin D forest plot. **(A)** HOMA-IR, **(B)** TC, **(C)** TG, **(D)** LDL-c, **(E)** hs-CRP, **(F)** LH, **(G)** Regular menstrual cycles, **(H)** Total testosterone.

The umbrella meta-analysis included 10 studies reporting on Vitamin D (43–52). A total of 29 relevant outcomes were recorded, with the experimental group receiving oral Vitamin D and the control group receiving a placebo. Notably, HOMA-IR (SMD: 0.09; 95% CI: −0.38, 0.56; *I*^2^ = 92.4%, *p* < 0.001), TC (SMD: −0.55; 95% CI: −0.82, −0.27; *I*^2^ = 96.2%, *p* < 0.001), TG (SMD: −1.02; 95% CI: −2.14, 0.10; *I*^2^ = 93.0%, *p* < 0.001), LDL-c (SMD: −2.62; 95% CI: −4.27, −0.97; *I*^2^ = 95.1%, *p* < 0.001), hs-CRP (SMD: -0.74; 95% CI: −1.10, −0.39; *I*^2^ = 44.9%, *p* = 0.178), LH (SMD: −0.41; 95% CI: −0.54, −0.28; *I*^2^ = 0.0%, *p* = 0.927), regular menstrual cycles (SMD: 1.38; 95% CI: 1.21, 1.56; *I*^2^ = 0.0%, *p* = 0.583), and total testosterone (SMD: −0.04; 95% CI: −0.18, 0.10; *I*^2^ = 69.1%, *p* = 0.072) were among the outcomes assessed (Figure 7). Given the significant high heterogeneity observed in the related outcomes, sensitivity analyses were conducted (Supplementary Figure S4). After excluding studies with considerable heterogeneity, HOMA-IR (SMD: −0.14; 95% CI: −0.66, 0.37; *I*^2^ = 80.0%, *p* = 0.026), TC (SMD: −11.28; 95% CI: −13.36, −9.21; *I*^2^ = 0.0%, *p* = 0.873), and TG (SMD: −8.99; 95% CI: −12.10, −5.89; *I*^2^ = 14.1%, *p* = 0.312) were re-evaluated (Supplementary Figure S4).

##### 3.2.5.2 Vitamin E

**Figure 8:**
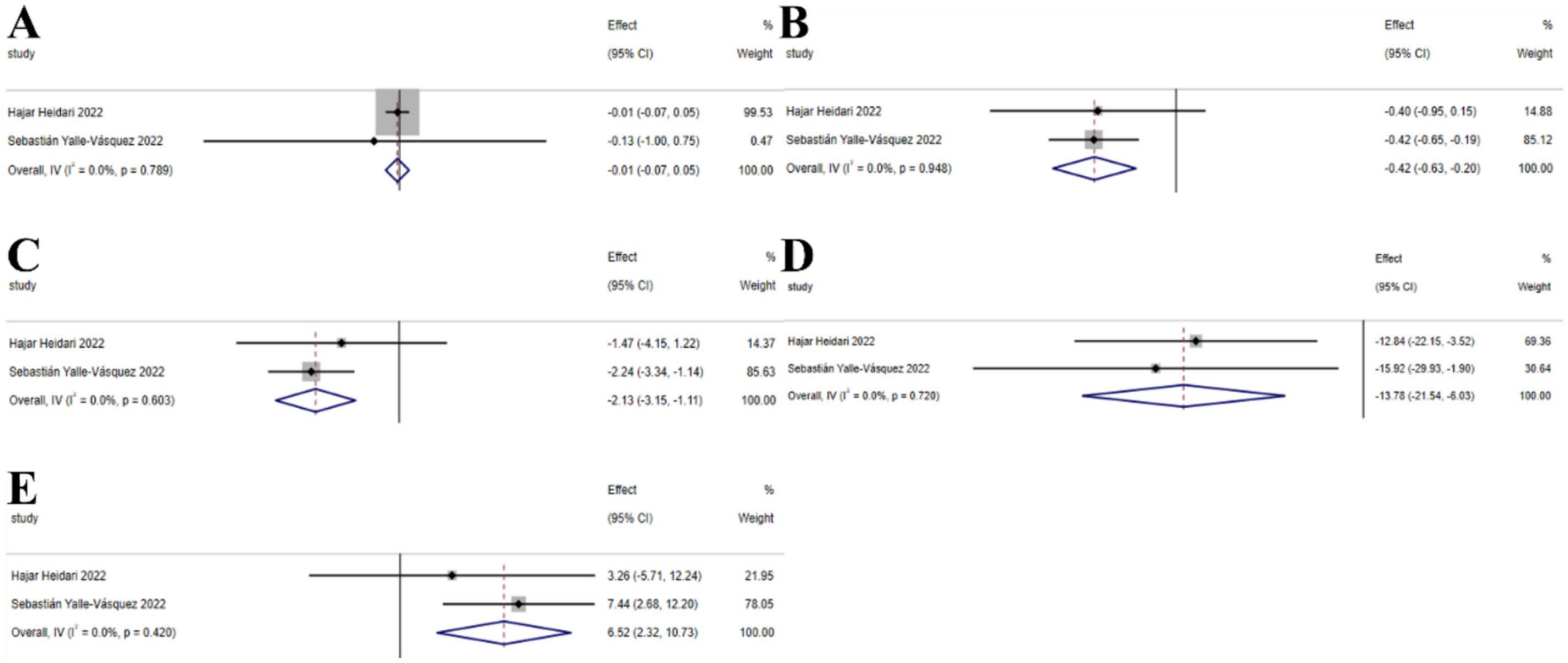
Vitamin E forest plot. **(A)** BMI, **(B)** HOMA-IR, **(C)** Insulin, **(D)** LDL-c, **(E)** SHBG.

The umbrella meta-analysis included 2 studies reporting on Vitamin E (53, 54). A total of 26 relevant outcomes were recorded, with the experimental group receiving oral Vitamin E and the control group receiving a placebo. Notably, BMI (SMD: −0.01; 95% CI: −0.07, 0.05; *I*^2^ = 0.0%, *p* = 0.789), HOMA-IR (SMD: −0.42; 95% CI: −0.63, −0.20; *I*^2^ = 0.0%, *p* = 0.948), insulin (SMD: −2.13; 95% CI: −3.15, −1.11; *I*^2^ = 0.0%, *p* = 0.603), LDL-c (SMD: −13.78; 95% CI: −21.54, −6.03; *I*^2^ = 0.0%, *p* = 0.720), and SHBG (SMD: 6.52; 95% CI: 2.32, 10.73; *I*^2^ = 0.0%, *p* = 0.420) were among the outcomes assessed (Figure 8).

#### 3.2.6 Probiotics

**Figure 9:**
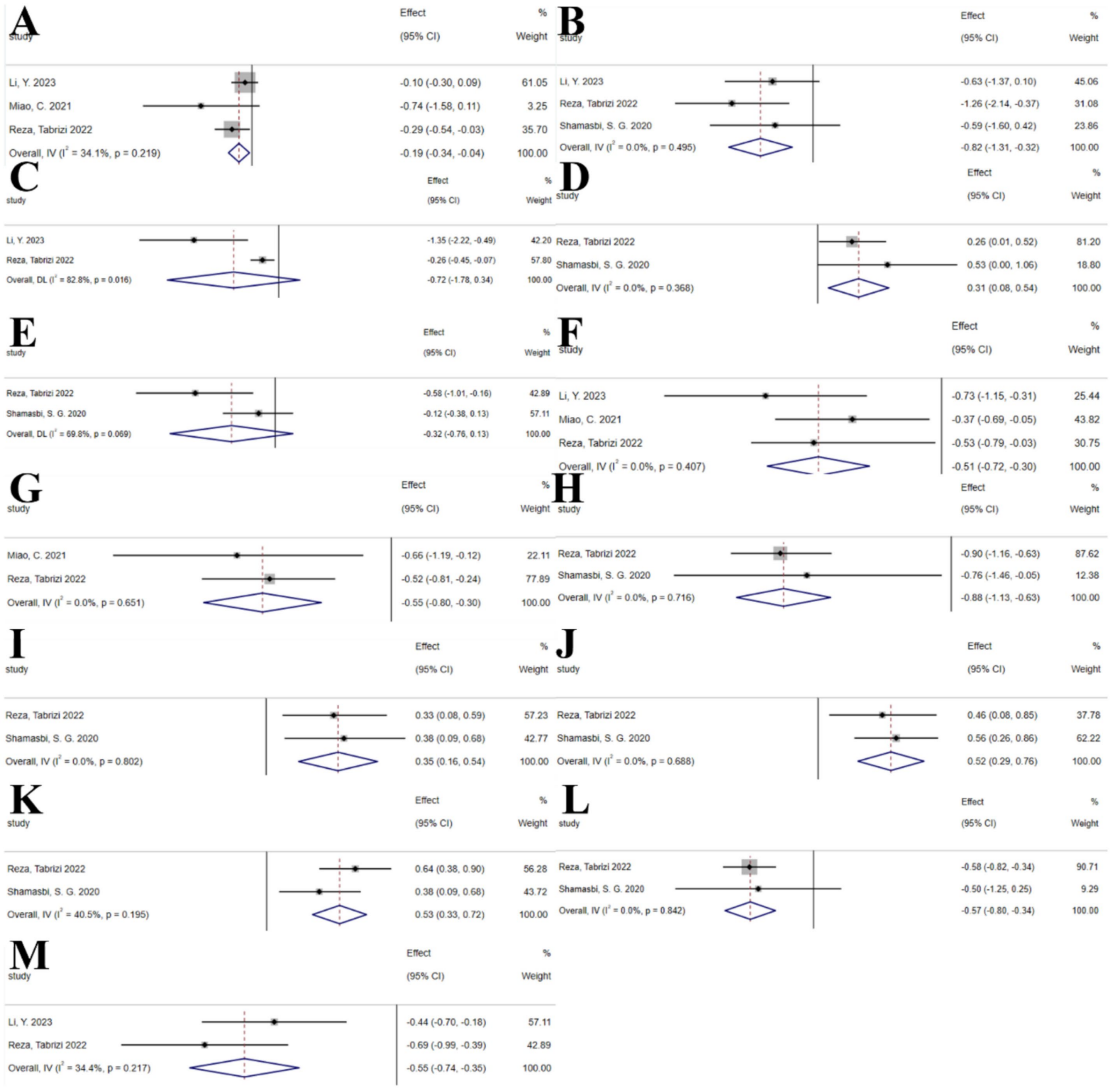
Probiotics forest plot. **(A)** BMI, **(B)** CRP, **(C)** FPG, **(D)** GSH, **(E)** Hirsutism, **(F)** HOMA-IR, **(G)** Insulin, **(H)** Malondialdehyde, **(I)** NO, **(J)** SHBG, **(K)** TAC, **(L)** Total testosterone levels, **(M)** vLDL-C.

The umbrella meta-analysis included 4 studies reporting on probiotics (55–58). A total of 24 relevant outcomes were recorded, with the experimental group receiving oral probiotics and the control group receiving a placebo. Notably, BMI (SMD: −0.19; 95% CI: −0.34, −0.04; *I*^2^ = 34.1%, *p* = 0.219), CRP (SMD: −0.82; 95% CI: −1.31, −0.32; *I*^2^ = 0.0%, *p* = 0.495), FPG (SMD: −0.72; 95% CI: −1.78, 0.34; *I*^2^ = 82.8%, *p* = 0.016), GSH (SMD: 0.31; 95% CI: 0.08, 0.54; *I*^2^ = 0.0%, *p* = 0.368), hirsutism (SMD: −0.32; 95% CI: −0.76, 0.13; *I*^2^ = 69.8%, *p* = 0.069), HOMA-IR (SMD: −0.51; 95% CI: −0.72, −0.30; *I*^2^ = 0.0%, *p* = 0.407), insulin (SMD: −0.55; 95% CI: −0.80, −0.30; *I*^2^ = 0.0%, *p* = 0.651), MDA (SMD: −0.88; 95% CI: −1.13, −0.63; *I*^2^ = 0.0%, *p* = 0.716), NO (SMD: 0.35; 95% CI: 0.16, 0.54; *I*^2^ = 0.0%, *p* = 0.802), SHBG (SMD: 0.52; 95% CI: 0.29, 0.76; *I*^2^ = 0.0%, *p* = 0.688), TAC (SMD: 0.53; 95% CI: 0.33, 0.72; *I*^2^ = 40.5%, *p* = 0.195), total testosterone (SMD: −0.57; 95% CI: −0.80, −0.34; I^2^ = 0.0%, *p* = 0.842), and vLDL-C (SMD: −0.55; 95% CI: −0.74, −0.35; *I*^2^ = 34.4%, *p* = 0.217) were among the outcomes assessed (Figure 9).

#### 3.2.7 Other indicators

In this umbrella review, indicators reported by only a single umbrella meta-analysis could not be subjected to traditional umbrella meta-analysis to assess their effect sizes and consistency (59–62). However, the reporting of these indicators provides insights into potentially overlooked aspects of PCOS treatment with nutritional supplements. Appendix B records the relevant indicators.

#### 3.2.8 Bibliometric analysis

**Figure 10:**
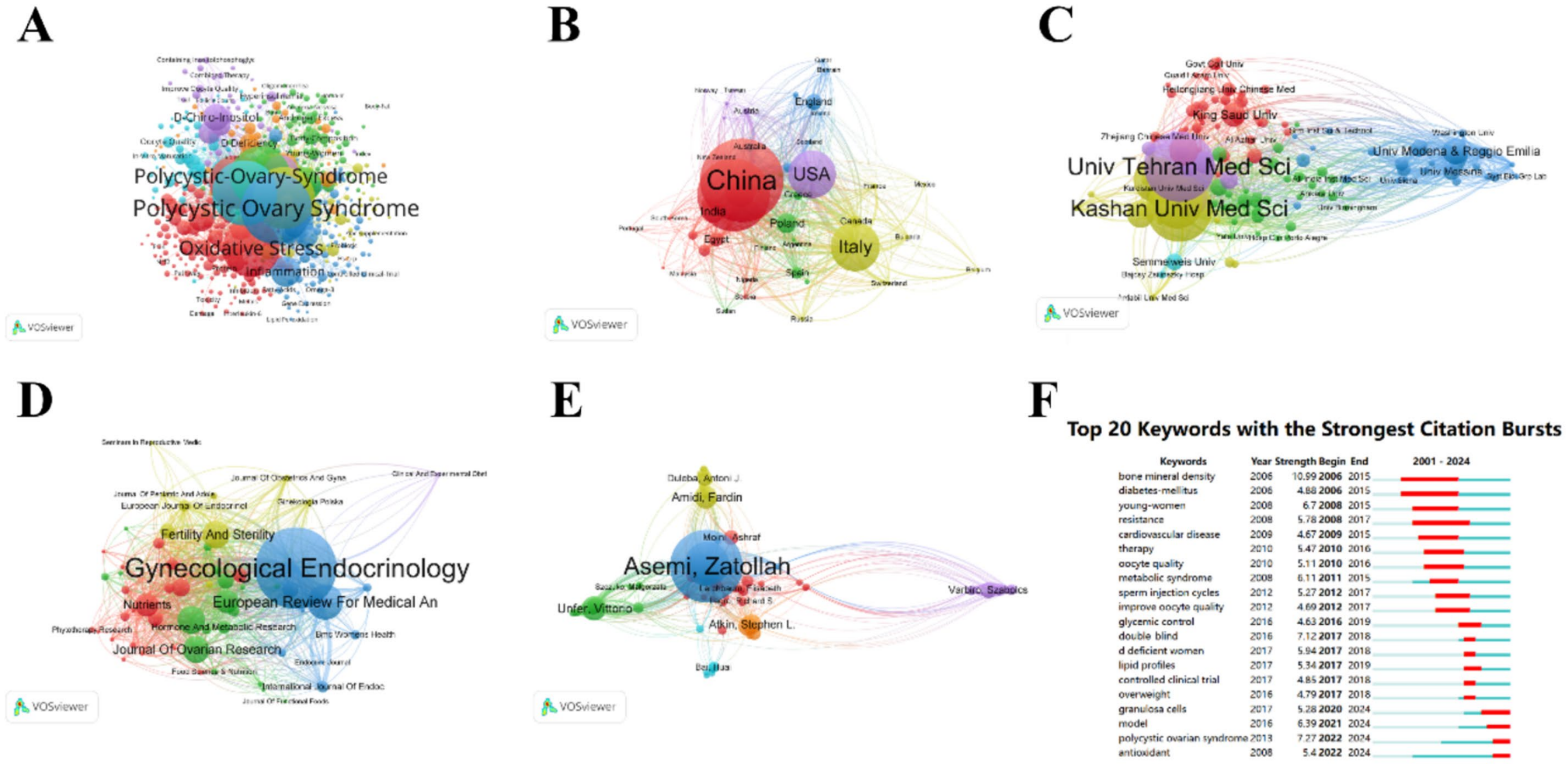
Visualization. **(A)** Key words, **(B)** Country, **(C)** Institution, **(D)** Periodical, **(E)** Author, **(F)** Top 20 Keyword Bursts. In the map, larger nodes represent a higher number of publications. The thickness of the connecting lines indicates the intensity of collaboration, with thicker lines signifying more frequent joint publications. Nodes of different colors represent different clusters.

The therapeutic potential of nutritional supplements for PCOS is gaining increasing recognition, with a growing body of literature emphasizing their clinical application. To assess the scientific trajectory of this field, a comprehensive bibliometric analysis was conducted using the WoS database from January 1, 2001, to September 13, 2024. The search strategy is recorded in Appendix A. The search yielded 2,345 articles, with only 1,468 experimental papers included. These articles were cited a total of 35,989 times, with an average of 24.52 citations per paper. Quantitative and visual analysis was performed using VOSviewer. Among 464 journals, 58 published 5 or more articles; among 7,352 authors, 82 published 5 or more articles; among 1,941 institutions, 135 published 5 or more articles; among 78 countries, 48 published 5 or more articles; among 5,016 keywords, 538 appeared 5 or more times (Figure 10). Table 1 lists the top 10 most-cited articles. The surge in citations for different keywords from 2006 to 2024 reflects the evolution of research hotspots in PCOS. It is evident that the focus on nutritional supplements has gradually shifted from early cell models to current clinical trials. However, to date, individualized dosing regimens for nutritional supplements in the treatment of PCOS have not been clearly defined. In the future, nutritional supplements will likely place greater emphasis on individualized plans to achieve optimal outcomes (63).

**Table 1:** The top 10 cited publications.

Publication	Descriptions	Citation					Average per year	Total	First author
		2020	2021	2022	2023	2024			
Prevalence, phenotype and cardiometabolic risk of polycystic ovary syndrome under different diagnostic criteria	Prevalence, phenotype, and cardiometabolic risk of PCOS according to different diagnostic criteria.	41	55	52	54	26	35.69	464	Yildiz, BO
The role of free radicals and antioxidants in reproduction	The role of free radicals and antioxidants in reproduction may have an important impact on the oxidative stress state of PCOS patients.	18	39	16	12	7	17.21	327	Agarwal, A
Role of insulin resistance in endothelial dysfunction	The role of insulin resistance in endothelial dysfunction, which may be associated with increased risk of cardiovascular disease associated with PCOS.	43	33	32	21	32	27.17	326	Muniyappa, R
Functional Hypothalamic Amenorrhea: An Endocrine Society Clinical Practice Guideline	Endocrine Society Clinical Practice Guidelines for Functional Hypothalamic Amenorrhea Provide Guidance for the Diagnosis and Treatment of Patients with PCOS	31	42	57	45	37	32.63	261	Gordon, CM
Position statement: Glucose intolerance in polycystic ovary syndrome – A position statement of the androgen excess society	The positional statement of abnormal glucose tolerance in polycystic ovary syndrome provides a new perspective on blood glucose management in PCOS patients.	8	12	9	5	6	13.83	249	Salley, KES
The effects of a whole grain-enriched hypocaloric diet on cardiovascular disease risk factors in men and women with metabolic syndrome	The effect of a low-calorie diet rich in whole grains on cardiovascular disease risk factors in patients with metabolic syndrome may have guiding significance for lifestyle intervention in PCOS patients.	13	9	6	2	3	13.12	223	Katcher, HI
Diet and lifestyle in the prevention of ovulatory disorder infertility	The role of diet and lifestyle in preventing anovulatory infertility may help PCOS patients adjust their diet and lifestyle.	13	20	25	27	18	12.33	222	Chavarro, JE
Low serum 25-hydroxyvitamin D concentrations are associated with insulin resistance and obesity in women with polycystic ovary syndrome	Low serum 25-hydroxyvitamin D concentrations are associated with insulin resistance and obesity in women with PCOS, suggesting that vitamin D may play a role in PCOS treatment.	9	15	14	12	7	11.32	215	Hahn, S
Mass spectrometry and immunoassay: how to measure steroid hormones today and tomorrow	Mass spectrometry and immunoassays: How to measure steroid hormones today and in the future, providing new technical means for measuring hormone levels in PCOS patients.	23	30	22	33	15	20.40	204	Taylor, AE
Androgen Therapy in Women: A Reappraisal: An Endocrine Society Clinical Practice Guideline	Reassessment of androgen therapy in women: The Endocrine Society’s clinical practice guideline provides guidance on androgen therapy in patients with PCOS.	18	29	21	11	17	17.91	197	Wierman, ME

## 4 Discussion

Previous research has suggested that nutritional supplement therapy may be a promising treatment strategy (64). We conducted a comprehensive search on nutritional supplements, including a total of 45 studies encompassing umbrella meta-analyses on the treatment of PCOS with n-3 polyunsaturated fatty acids, α-lipoic acid, plant polyphenols (curcumin, green tea, resveratrol), minerals (chromium, selenium), melatonin supplements, vitamins, inositol, probiotics, and L-carnitine. Our results indicate that n-3 polyunsaturated fatty acids, inositol, plant polyphenols (curcumin and green tea), minerals (chromium and selenium), vitamins D and E, and probiotics all show potential benefits in improving the metabolic and reproductive health of PCOS patients. The keyword burst plot in the bibliometric analysis (Figure 10E) indicates that the early research focus, as shown by the burst of keywords such as “bone mineral density,” “diabetes mellitus,” and “young women,” has shifted toward the antioxidant and personalized aspects of nutritional supplements in recent years, as indicated by the burst of keywords like “polycystic ovarian syndrome” and “antioxidant” (65–67).

Our results align with existing evidence that nutritional supplements ameliorate insulin resistance, lipid profiles, and hormonal imbalances in PCOS (68, 69). n-3 Polyunsaturated fatty acids significantly improved the adiponectin levels, BMI, fasting blood glucose, HDL-c, HOMA-IR, LDL-C, MDA, SHBG, TG, and total cholesterol in PCOS patients, but not FSH and total testosterone levels. Studies by Yuan et al. (27) and Zhou et al. (29) both indicated that supplementation with n-3 polyunsaturated fatty acids had no significant effect on FSH levels, which is consistent with the previous report by Salek et al. (70), indicating a lack of comprehensive evidence specifically targeting FSH improvement. The studies by Hajishafiee et al. (23) and Zeng and Yang et al. (30) both indicated that supplementation with n-3 polyunsaturated fatty acids had no significant effect on total testosterone levels, which is not consistent with the results of the study by Albardan et al. (71). In the study by Hajishafiee, et al. (23), most clinical trials used fish oil or flaxseed oil; in Hajishafiee 2016’s study, different types of n-3 polyunsaturated fatty acids were used, including EPA and DHA from fish oil and plant-derived ALA (such as ALA from flaxseed oil and walnuts). Different doses and types of n-3 PUFA may have inconsistent effects on testosterone metabolic parameters (72). Inositol improved androstenedione, HOMA-IR, LH, SHBG, BMI, fasting blood glucose, fasting insulin, FSH, and pregnancy rates, which is consistent with the study by Gudović et al. (73). This is because inositol improves insulin resistance (IR) by promoting the translocation of glucose transporter (GLUT4), enhancing cellular glucose uptake, thereby lowering fasting insulin levels (74) and regulating the signaling of FSH and LH, improving the LH/FSH ratio, reducing androgen levels, and thus alleviating hyperandrogenemia (75). Curcumin significantly improved BMI, fasting blood glucose, fasting insulin, HDL-C, HOMA-IR, insulin, QUICK, SHBG, TC, total cholesterol, total testosterone, waist circumference, and WHR in PCOS patients, but had no significant effect on gastrointestinal adverse events, postprandial blood glucose, or pregnancy rates. Curcumin has significant anti-inflammatory effects, mainly by downregulating the NLRP3 inflammasome, and regulating NF-κB signaling and interleukin secretion are the most prominent functional mechanisms of curcumin in modulating the inflammasome (76), thus potentially focusing more on improving the inflammatory levels in PCOS patients. The study by Yongwatana et al. (77) suggests that curcumin may have a positive effect on gastrointestinal symptoms, but whether it significantly affects gastrointestinal adverse events in PCOS patients requires further research to confirm. Green tea can improve BMI in PCOS patients, and the health benefits of green tea are attributed to its natural antioxidants, namely catechins, especially epigallocatechin-3-gallate (EGCG), which has beneficial effects on health, including high antioxidant, bone protection, neuroprotection, anticancer, antihyperlipidemic, and antidiabetic effects (78). Regarding minerals, our results show no significant improvement in FBS, FG, FSI, HOMA-IR, or SHBG in PCOS patients. This is consistent with the conclusion by Pokorska-Niewiada et al. (79), suggesting that a more comprehensive treatment approach, including lifestyle changes, pharmacological treatment, and possibly nutritional supplements, rather than relying solely on mineral supplements, may be needed for PCOS patients. Our results indicate that vitamin D does not significantly affect HOMA-IR, TG, or total testosterone, which is contrary to the studies by Miao et al. (51) and Kiani et al. (68), and may be related to the dose and treatment duration of vitamin D (80). Vitamin E does not significantly affect BMI, with one study reporting a small but significant decrease in waist circumference (*d* = 0.3), but no significant change in weight (81). Probiotics significantly improved BMI, CRP, GSH, HOMA-IR, insulin, MDA, NO, SHBG, TAC, total testosterone, and vLDL-C in PCOS patients, which is consistent with the study by Angoorani et al. (82). We found that probiotics did not significantly improve hirsutism in PCOS patients, which is consistent with the study by Wu et al. (83).

We assessed the methodological quality of the included meta-analyses using the AMSTAR 2 checklist. Among the 46 studies, 21 (45.7%) were rated as high quality, 13 (28.3%) as medium quality, and 12 (26.1%) as low quality. The overall fair-to-good quality of the included studies reinforces the reliability of our umbrella review findings. The detailed item-by-item quality assessment results for each study are presented in Supplementary Table S1.

In this study, there was heterogeneity among different umbrella meta-analyses. The persistent heterogeneity, even after sensitivity analyses, underscores the influence of factors like dosing regimens and supplement formulations. Despite significant overall effects observed in previous umbrella meta-analyses, there are still some controversies. The differences in results can be attributed to different treatment doses and durations, different types of analyses, different umbrella meta-analysis qualities, and different sample sizes. For example, in the case of probiotics, except for the synbiotic pomegranate juice used in the study by (88), other studies used probiotic capsules, and the strains of probiotics in each study were not consistent. For instance, the probiotic capsule used in the study by (84, 89) included *Lactobacillus casei*, *Lactobacillus acidophilus*, *Streptococcus thermophilus*, *Lactobacillus bulgaricus*, *Bifidobacterium longum*, *Bifidobacterium breve*, and *Streptococcus thermophilus* (each strain at a concentration of 7 × 10^9 CFU/g), while the probiotic capsule used in the study by (85, 90) included *Lactobacillus acidophilus*, *Lactobacillus rhamnosus*, *Lactobacillus fermentum*, and *Bifidobacterium* (each strain at a concentration of 2 × 10^9 CFU/g), plus 200 micrograms of selenium.

**Figure 11:**
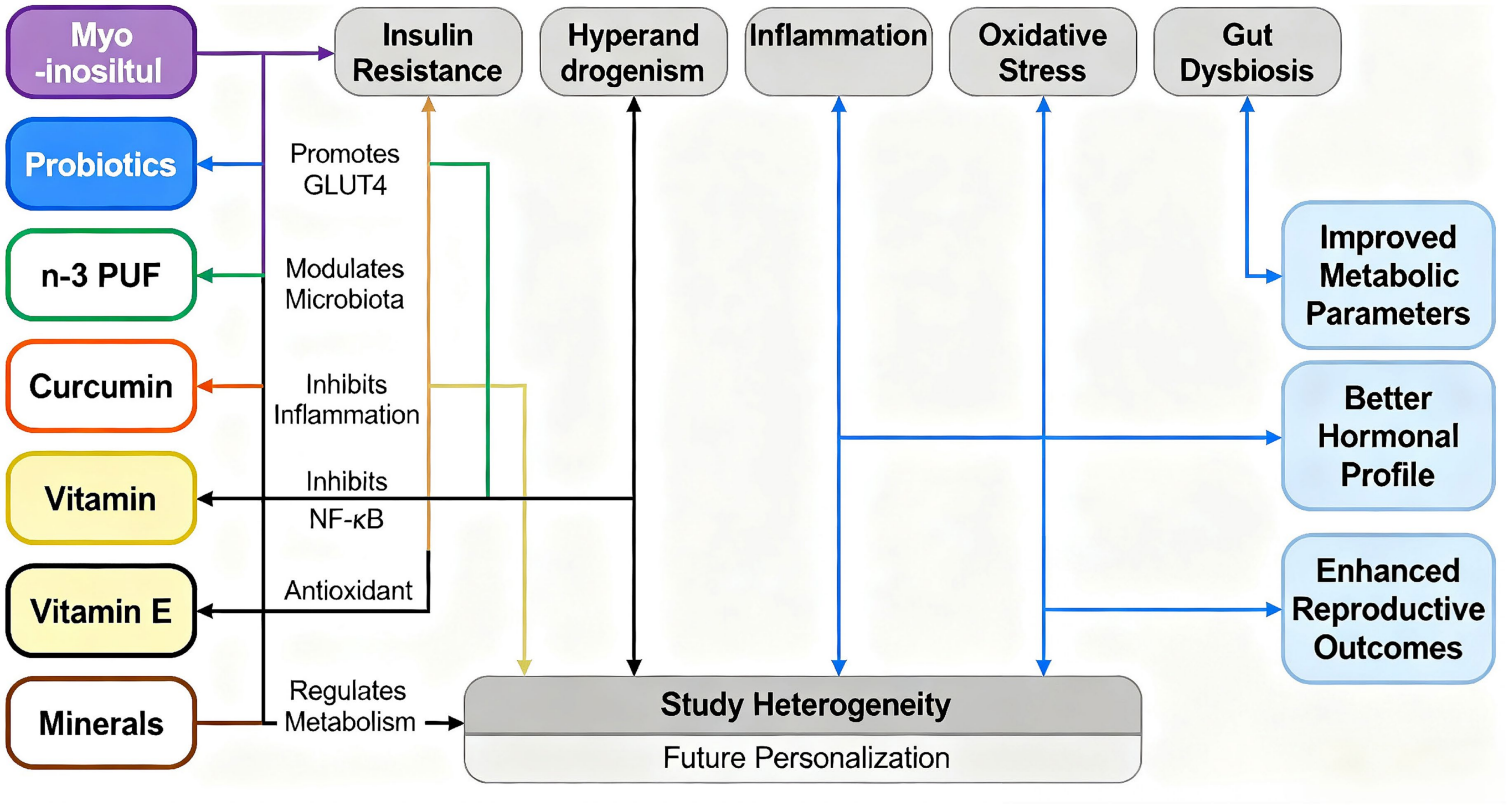
Schematic diagram illustrating the mechanism by which nutritional supplements improve metabolic and reproductive dysfunction. This figure summarizes the potential mechanisms by which various nutritional supplements (such as inositol, probiotics, n-3 polyunsaturated fatty acids, curcumin, vitamins and minerals) exert beneficial effects. These supplements target core pathological links such as insulin resistance, hyperandrogenemia, inflammation, oxidative stress and dysbiosis of the intestinal flora (e.g., polycystic ovary syndrome, PCOS). By regulating these key pathways, these interventions can work synergistically to regulate metabolism and ultimately achieve the effects of improving metabolic parameters, optimizing hormone levels and enhancing reproductive health outcomes. This figure also highlights the heterogeneity in current research and points to the direction of future personalized nutritional therapy.

This study is the first comprehensive analysis of the impact of nutritional supplements on the treatment of PCOS. We adhered to the Preferred Reporting Items for Systematic Reviews and Umbrella meta-analyses (PRISMA) guidelines, enhancing the transparency and reproducibility of our research. We have clarified that inositol and probiotics have significant outcomes in the treatment of PCOS. Concurrently, bibliometric analysis indicates a shift toward the study of the antioxidant aspects of nutritional supplements, aligning with significant advancements in research on inositol and probiotics (84, 85). A study discusses the importance of personalized diets in the treatment of PCOS, emphasizing the relationship between healthy eating, physiological homeostasis regulation, and disease recovery (86). Relevant research suggests that individual genetic variations affect metabolic responses to diet, highlighting the importance of integrating nutrigenomics with nutritional supplementation in formulating personalized dietary guidelines (86). Szczuko et al. (63) emphasize that the clinical phenotypes of PCOS can change over the life cycle and can coexist within the same patient. While personalized treatment remains the primary approach, phenotypic grouping and adherence to treatment recommendations may also be clinically applicable. Precise recommendations should be implemented well before the onset of metabolic complications, which is particularly crucial for women with PCOS (63). Cowan et al. (87) suggest that self-management strategies support lifestyle changes in PCOS patients and increase interaction with qualified health professionals, thereby enhancing treatment outcomes. As demonstrated by our bibliometric research, the treatment of PCOS with nutritional supplements is increasingly trending toward personalization in the future (Figure 11).

## 5 Conclusion

Nutritional supplements show promise for improving metabolic and reproductive outcomes in PCOS, but heterogeneity highlights the need for personalized approaches and further validation. Future research should focus on personalized treatment regimens and the antioxidant properties of nutritional supplements to achieve optimal therapeutic outcomes for PCOS.

## Data Availability

The original contributions presented in the study are included in the article/Supplementary material, further inquiries can be directed to the corresponding authors.

## Glossary

AMSTAR: A MeaSurement Tool to Assess systematic Review
BMI: Body Mass Index
CI: Confidence Interval
CRP: C-reactive Protein
DHA: Docosahexaenoic Acid
EPA: Eicosapentaenoic Acid
FBG: Fasting Blood Glucose
FG: Fasting Glucose
FI: Fasting Insulin
FSH: Follicle-Stimulating Hormone
GSH: Glutathione
HDL-C: High-Density Lipoprotein Cholesterol
HOMA-IR: Homeostatic Model Assessment of Insulin Resistance
hs-CRP: high-sensitivity C-reactive Protein
LDL-C: Low-Density Lipoprotein Cholesterol
LH: Luteinizing Hormone
MDA: Malondialdehyde
MD: Mean Difference
NO: Nitric Oxide
OR: Odds Ratio
PCOS: Polycystic Ovary Syndrome
PRISMA: Preferred Reporting Items for Systematic Reviews and Meta-Analyses
QUICKI: Quantitative Insulin Sensitivity Check Index
RCT: Randomized Controlled Trial
RR: Risk Ratio
SHBG: Sex Hormone-Binding Globulin
SMD: Standardized Mean Difference
TAC: Total Antioxidant Capacity
TC: Total Cholesterol
TG: Triglycerides
vLDL-C: very Low-Density Lipoprotein Cholesterol
WHR: Waist-to-Hip Ratio
