# Supplementary material for: Efficacy of dietary supplements as an adjunctive therapy for polycystic ovary syndrome: an umbrella meta-analysis
Source: Front Nutr. 2025 Oct 29;12:1705284. doi: 10.3389/fnut.2025.1705284 (PMC12605168; doi:10.3389/fnut.2025.1705284)
Supplement: Supplementary file 1 [file Table_1.docx]

**Supplementary Table S1** The results of quality assessment included umbrella meta-analyses based on AMSTAR2 questionnaire.

| Study | Q1 | Q2 | Q3 | Q4 | Q5 | Q6 | Q7 | Q8 | Q9 | Q10 | Q11 | Q12 | Q13 | Q14 | Q15 | Q16 | Overall |
| --- | --- | --- | --- | --- | --- | --- | --- | --- | --- | --- | --- | --- | --- | --- | --- | --- | --- |
| *Arentz, S 2017* | √ | × | × | √ | √ | √ | × | √ | × | √ | √ | √ | √ | √ | × | √ | **Moderate** |
| *Hajishafiee, M 2016* | √ | × | × | √ | √ | √ | × | √ | × | √ | √ | √ | √ | √ | × | √ | **Moderate** |
| *Sadeghi, A 2017* | × | × | × | √ | √ | √ | √ | √ | √ | √ | √ | √ | √ | √ | √ | √ | **High** |
| *Tosatti, J. A. G 2021* | × | × | × | √ | √ | √ | √ | √ | √ | √ | × | × | × | × | × | × | **Low** |
| *Xia, Y 2021* | × | × | × | √ | √ | √ | √ | √ | √ | √ | × | × | × | × | × | × | **Low** |
| *Yang, K 2018* | × | × | × | √ | √ | √ | √ | √ | √ | √ | × | × | × | × | × | × | **Low** |
| *Yuan, J 2021* | × | × | × | √ | √ | √ | √ | √ | √ | √ | × | × | × | × | × | × | **Low** |
| *Zhou, J 2023* | √ | √ | √ | √ | √ | √ | √ | √ | √ | √ | √ | √ | √ | √ | √ | √ | **High** |
| *Abu-Zaid, A 2024* | √ | √ | √ | √ | √ | √ | √ | √ | √ | √ | √ | √ | √ | √ | × | × | **Moderate** |
| *Fadlalmola 2023* | √ | √ | √ | √ | √ | √ | √ | √ | √ | √ | √ | √ | √ | √ | × | × | **High** |
| *Balieiro 2024* | √ | √ | √ | √ | √ | √ | √ | √ | √ | √ | √ | √ | √ | √ | × | × | **High** |
| *Lesani 2022* | √ | √ | √ | √ | √ | √ | √ | √ | √ | √ | √ | √ | √ | × | × | × | **Moderate** |
| *Ahmed Abu-Zaid 2023* | √ | √ | √ | √ | √ | √ | √ | √ | √ | √ | √ | √ | √ | √ | √ | √ | **High** |
| *Pei-yu Wu 2022* | √ | √ | √ | √ | √ | √ | √ | √ | √ | √ | √ | √ | √ | √ | √ | √ | **High** |
| *Javad Heshmati 2018* | √ | √ | √ | √ | √ | √ | √ | √ | √ | √ | √ | √ | √ | √ | × | √ | **Moderate** |
| *Siavash Fazelian 2017* | √ | √ | √ | √ | √ | √ | √ | √ | √ | √ | × | √ | √ | × | √ | √ | **Moderate** |
| *Ziaei 2024* | √ | √ | √ | √ | √ | √ | √ | √ | √ | √ | √ | × | √ | √ | √ | × | **High** |
| *Greff 2023* | √ | √ | √ | √ | √ | √ | √ | √ | √ | × | √ | √ | √ | √ | × | × | **High** |
| *Pundir 2017* | √ | √ | √ | √ | √ | √ | √ | √ | √ | × | √ | √ | √ | × | × | × | **Moderate** |
| *Unfer 2017* | √ | √ | √ | √ | √ | √ | √ | √ | √ | × | √ | √ | √ | √ | × | × | **High** |
| *Zeng 2018* | √ | √ | √ | √ | √ | √ | √ | √ | √ | × | √ | √ | √ | × | × | × | **Moderate** |
| *Abdelazeem 2022* | √ | √ | √ | √ | √ | √ | √ | √ | √ | √ | × | √ | √ | √ | × | √ | **High** |
| *Chien 2021* | √ | √ | √ | √ | √ | √ | √ | √ | √ | √ | × | √ | √ | √ | × | √ | **High** |
| *Simental-Mendía 2022* | √ | √ | √ | √ | √ | √ | √ | √ | √ | √ | √ | √ | √ | √ | × | √ | **High** |
| *Nouri 2022* | √ | √ | × | √ | √ | √ | √ | √ | √ | × | × | √ | √ | √ | √ | × | **Moderate** |
| *Shen 2022* | √ | √ | √ | √ | √ | √ | √ | √ | √ | √ | × | √ | √ | √ | × | √ | **High** |
| *Xie 2019* | √ | √ | √ | √ | √ | √ | √ | √ | √ | √ | × | √ | √ | √ | × | √ | **High** |
| *Hajar Heidari 2022* | √ | √ | √ | √ | √ | √ | √ | √ | √ | √ | √ | √ | √ | √ | √ | √ | **High** |
| *Sebastián Yalle-Vásquez 2022* | √ | √ | √ | √ | √ | √ | √ | √ | √ | × | √ | √ | √ | √ | × | √ | **Moderate** |
| *Akbari, M 2018* | √ | √ | √ | √ | √ | √ | √ | √ | √ | √ | √ | √ | √ | √ | × | × | **High** |
| *Fang, F 2017* | √ | √ | × | √ | √ | √ | √ | √ | √ | × | √ | √ | √ | √ | × | × | **Moderate** |
| *Gao, H 2021* | √ | √ | √ | √ | √ | √ | √ | √ | √ | √ | √ | √ | √ | √ | × | × | **High** |
| *Han, Y 2023* | √ | √ | √ | √ | √ | √ | √ | √ | √ | √ | √ | √ | √ | √ | × | × | **High** |
| *Jin, B 2020* | √ | √ | √ | √ | √ | √ | √ | √ | √ | √ | √ | √ | √ | √ | × | × | **High** |
| *Luo, J 2021* | √ | √ | √ | √ | √ | √ | √ | √ | √ | √ | √ | √ | √ | √ | × | × | **High** |
| *Miao, C. Y 2019* | √ | √ | √ | √ | √ | √ | √ | √ | √ | √ | √ | √ | √ | √ | × | × | **High** |
| *Pergialiotis, V 2017* | √ | √ | √ | √ | √ | √ | √ | √ | √ | √ | √ | √ | √ | √ | × | × | **High** |
| *Xue, Y 2017* | √ | √ | × | √ | √ | √ | √ | √ | √ | √ | √ | √ | × | √ | × | √ | **Moderate** |
| *Zhang, B 2023* | √ | √ | √ | √ | √ | √ | √ | √ | √ | √ | √ | √ | √ | √ | √ | √ | **High** |
| *Li, Y 2023* | √ | √ | √ | √ | √ | √ | √ | √ | √ | √ | √ | √ | √ | √ | √ | × | **High** |
| *Miao, C 2021* | √ | √ | √ | √ | √ | √ | √ | √ | × | √ | √ | √ | √ | × | √ | × | **Moderate** |
| *Reza, Tabrizi 2022* | √ | √ | √ | √ | √ | √ | √ | √ | √ | √ | √ | √ | √ | × | √ | × | **High** |
| *Shamasbi, S G. 2020* | √ | √ | √ | √ | √ | √ | √ | √ | × | √ | √ | √ | √ | × | √ | × | **Moderate** |
| *Yuling, Li 2023* | √ | √ | √ | √ | √ | √ | √ | √ | √ | √ | √ | √ | √ | × | √ | × | **High** |
| *Gong, Y 2023* | √ | √ | √ | √ | √ | √ | √ | √ | √ | √ | √ | √ | × | √ | × | √ | **Moderate** |
| *Shukri, M. F 2022* | √ | √ | × | √ | √ | √ | √ | √ | √ | √ | √ | √ | × | √ | × | √ | **Low** |

**Note:**

1- Did the research questions and inclusion criteria for the review include the components of PICO?

2- Did the report of the review contain an explicit statement that the review methods were established prior to the conduct of the review, and did the report justify any significant deviations from the protocol?

3- Did the review authors explain their selection of the study designs for inclusion in the review?

4- Did the review authors use a comprehensive literature search strategy?

5- Did the review authors perform study selection in duplicate?

6- Did the review authors perform data extraction in duplicate?

7- Did the review authors provide a list of excluded studies and justify the exclusions?

8- Did the review authors describe the included studies in adequate detail?

9- Did the review authors use a satisfactory technique for assessing the risk of bias (RoB) in individual studies that were included in the review?

10- Did the review authors report on the sources of funding for the studies included in the review?

11- If umbrella meta-analysis was performed, did the review authors use appropriate methods for the statistical combination of results?

12- If an umbrella meta-analysis was performed, did the review authors assess the potential impact of RoB in individual studies on the results of the umbrella meta-analysis or other evidence synthesis?

13- Did the review authors account for RoB in individual studies when interpreting/ discussing the review results?

14- Did the review authors provide a satisfactory explanation for and discussion of any heterogeneity observed in the review results?

15- If they performed quantitative synthesis, did the review authors conduct an adequate investigation of publication bias (small-study bias) and discuss its likely impact on the review results?

16- Did the review authors report any potential sources of conflict of interest, including any funding they received for conducting the review?
