# Supplementary material for: Efficacy of dietary supplements as an adjunctive therapy for polycystic ovary syndrome: an umbrella meta-analysis
Source: Front Nutr. 2025 Oct 29;12:1705284. doi: 10.3389/fnut.2025.1705284 (PMC12605168; doi:10.3389/fnut.2025.1705284)
Supplement: Supplementary file 2 [file Table_2.docx]

**Appendix 1. Pubmed search strategy**

Searched September 13, 2024

#1 ((((((((((((((((((((((((((((((((((((((((((((((((((((Inositol[MeSH Terms]) OR (Inositol[Title/Abstract])) OR (Chiro-Inositol[Title/Abstract])) OR (Chiro Inositol[Title/Abstract])) OR (Mesoinositol[Title/Abstract])) OR (Myoinositol[Title/Abstract])) OR (Fish Oils[MeSH Terms])) OR (Fish Oils[Title/Abstract])) OR (Liver Oils, Fish[Title/Abstract])) OR (Vitamin D[MeSH Terms])) OR (Vitamin D[Title/Abstract])) OR (Minerals[MeSH Terms])) OR (Minerals[Title/Abstract])) OR (Magnesium[Title/Abstract])) OR (Probiotics[MeSH Terms])) OR (Probiotics[Title/Abstract])) OR (Probiotic[Title/Abstract])) OR (Antioxidants[Title/Abstract])) OR (Antioxidant[Title/Abstract])) OR (Anti-Oxidant[Title/Abstract])) OR (Anti-Oxidants[Title/Abstract])) OR (Zinc[MeSH Terms])) OR (Zinc[Title/Abstract])) OR (Vitamin B12[MeSH Terms])) OR (Vitamin B12[Title/Abstract])) OR (Cyanocobalamin[Title/Abstract])) OR (Folic Acid[MeSH Terms])) OR (Folic Acid[Title/Abstract])) OR (Pteroylglutamic Acid[Title/Abstract])) OR (Vitamin B9[Title/Abstract])) OR (Vitamin M[Title/Abstract])) OR (Coenzyme Q10[MeSH Terms])) OR (Coenzyme Q10[Title/Abstract])) OR (Ubiquinone[Title/Abstract])) OR (Coenzyme Q[Title/Abstract])) OR (Vitamin A[MeSH Terms])) OR (Vitamin A[Title/Abstract])) OR (Vitamin E[Title/Abstract])) OR (Vitamin E[MeSH Terms])) OR (Carnitine[MeSH Terms])) OR (Carnitine[Title/Abstract])) OR (Levocarnitine[Title/Abstract])) OR (Fatty Acids, Omega-3[MeSH Terms])) OR (Fatty Acids, Omega-3[Title/Abstract])) OR (N-3 Fatty Acid[Title/Abstract])) OR (Selenium[MeSH Terms])) OR (Selenium[Title/Abstract])) OR (Selenium-80[Title/Abstract])) OR (Resveratrol[MeSH Terms])) OR (Resveratrol[Title/Abstract])) OR (Curcumin[MeSH Terms])) OR (Curcumin[Title/Abstract])) OR (Curcumin Phytosome[Title/Abstract])  **(1,190,898)**

#2 (((Polycystic Ovary Syndrome[MeSH Terms]) OR (PCOS[MeSH Terms])) OR (Polycystic Ovary Syndrome[Title/Abstract])) OR (PCOS[Title/Abstract]) **(25,271)**

#3 ((meta-analysis[MeSH Terms]) OR (meta-analysis[Title/Abstract])) **(286,963)**

#4 #1 AND #2 AND #3 **(130)**

**Appendix 2. Web of Science search strategy**

Searched September 13, 2024

#1 (((((((((((((((((((((((((((((((((((((TS=(Inositol)) OR TS=(Chiro-Inositol)) OR TS=(Chiro-Inositol)) OR TS=(Mesoinositol)) OR TS=(Myoinositol)) OR TS=(Fish Oils)) OR TS=(Liver Oils, Fish)) OR TS=(Vitamin D)) OR TS=(Minerals)) OR TS=(Magnesium)) OR TS=(Probiotics)) OR TS=(Probiotic)) OR TS=(Antioxidants)) OR TS=(Antioxidant)) OR TS=(Anti-Oxidant)) OR TS=(Anti-Oxidants)) OR TS=(Zinc)) OR TS=(Vitamin B12)) OR TS=(Cyanocobalamin)) OR TS=(Folic Acid)) OR TS=(Pteroylglutamic Acid)) OR TS=(Vitamin B9)) OR TS=(Vitamin M)) OR TS=(Coenzyme Q10)) OR TS=(Ubiquinone)) OR TS=(Coenzyme Q10)) OR TS=(Vitamin A)) OR TS=(Vitamin E)) OR TS=(Carnitine)) OR TS=(Bicarnesine)) OR TS=(Levocarnitine)) OR TS=(Fatty Acids, Omega-3)) OR TS=(N-3 Fatty Acid)) OR TS=(Selenium)) OR TS=(Selenium-80)) OR TS=(Resveratrol)) OR TS=(Curcumin)) OR TS=(Curcumin Phytosome) **(6,600,702)**

#2 (TS=(Polycystic Ovary Syndrome)) OR TS=(PCOS) **(46,325)**

#3 (TS=(Meta-Analysis [Publication Type])) OR TS=(meta-analysis) **(457,748)**

#4 #1 AND #2 AND #3 **(280)**

**Appendix 3. Embase search strategy**

Searched September 13, 2024

#1 'inositol'/exp OR inositol:ab,ti OR 'chiro inositol':ab,ti OR mesoinositol:ab,ti OR myoinositol:ab,ti OR 'fish oil'/exp OR 'fish oils':ab,ti OR 'liver oils, fish':ab,ti OR 'vitamin d':ab,ti OR 'vitamin d'/exp OR 'minerals'/exp OR minerals:ab,ti OR magnesium:ab,ti OR 'probiotics'/exp OR probiotics:ab,ti OR antioxidants:ab,ti OR 'anti oxidant':ab,ti OR 'zinc'/exp OR zinc:ab,ti OR 'vitamin b12':ab,ti OR 'vitamin b12'/exp OR cyanocobalamin:ab,ti OR 'folic acid'/exp OR 'folic acid':ab,ti OR 'pteroylglutamic acid'/exp OR 'vitamin b9':ab,ti OR 'vitamin m':ab,ti OR 'coenzyme q10'/exp OR 'coenzyme q10':ab,ti OR ubiquinone:ab,ti OR 'coenzyme q':ab,ti OR 'vitamin a':ab,ti OR 'vitamin a'/exp OR 'vitamin e'/exp OR 'vitamin e':ab,ti OR 'carnitine'/exp OR carnitine:ab,ti OR bicarnesine:ab,ti OR levocarnitine:ab,ti OR 'fatty acids, omega-3'/exp OR 'fatty acids, omega-3':ab,ti OR 'n-3 fatty acid':ab,ti OR 'selenium'/exp OR selenium:ab,ti OR 'selenium 80':ab,ti OR resveratrol:ab,ti OR 'resveratrol'/exp OR 'curcumin'/exp OR curcumin:ab,ti OR 'curcumin phytosome':ab,ti **(1,171,432)**

#2 'ovary polycystic disease'/exp OR 'polycystic ovary syndrome':ab,ti OR pcos OR pcos:ab,ti **(44,830)**

#3 'meta analysis'/exp OR 'meta analysis':ab,ti **(417,058)**

#4 #1 AND #2 AND #3 **(227)**

**Appendix 4. Scopus search strategy**

Searched September 13, 2024

#1 ( TITLE-ABS-KEY ( meta-analysis ) ) AND ( ( TITLE-ABS-KEY ( polycystic AND ovary AND syndrome ) OR TITLE-ABS-KEY ( pcos ) ) ) AND ( ( TITLE-ABS-KEY ( inositol ) OR TITLE-ABS-KEY ( chiro-inositol ) OR TITLE-ABS-KEY ( mesoinositol ) OR TITLE-ABS-KEY ( myoinositol ) OR TITLE-ABS-KEY ( fish AND oils ) OR TITLE-ABS-KEY ( liver AND oils, AND fish ) OR TITLE-ABS-KEY ( vitamin AND d ) OR TITLE-ABS-KEY ( minerals ) OR TITLE-ABS-KEY ( magnesium ) OR TITLE-ABS-KEY ( probiotics ) OR TITLE-ABS-KEY ( probiotic ) OR TITLE-ABS-KEY ( antioxidants ) OR TITLE-ABS-KEY ( anti-oxidant ) OR TITLE-ABS-KEY ( zinc ) OR TITLE-ABS-KEY ( vitamin AND b12 ) OR TITLE-ABS-KEY ( cyanocobalamin ) OR TITLE-ABS-KEY ( folic AND acid ) OR TITLE-ABS-KEY ( pteroylglutamic AND acid ) OR TITLE-ABS-KEY ( vitamin AND b9 ) OR TITLE-ABS-KEY ( vitamin AND m ) OR TITLE-ABS-KEY ( coenzyme AND q10 ) OR TITLE-ABS-KEY ( ubiquinone ) OR TITLE-ABS-KEY ( coenzyme AND q ) OR TITLE-ABS-KEY ( vitamin AND a ) OR TITLE-ABS-KEY ( vitamin AND e ) OR TITLE-ABS-KEY ( carnitine ) OR TITLE-ABS-KEY ( bicarnesine ) OR TITLE-ABS-KEY ( levocarnitine ) OR TITLE-ABS-KEY ( fatty AND acids, AND omega-3 ) OR TITLE-ABS-KEY ( n-3 AND fatty AND acid ) OR TITLE-ABS-KEY ( selenium ) OR TITLE-ABS-KEY ( selenium-80 ) OR TITLE-ABS-KEY ( resveratrol ) OR TITLE-ABS-KEY ( curcumin ) OR TITLE-ABS-KEY ( curcumin AND phytosome ) ) ) **(248)**

**Appendix 5. Bibliometric Search Strategy**

Searched September 13, 2024

#1 (((((((((((((((((((((((((((((((((TS=(Inositol)) OR TS=(Chiro-Inositol)) OR TS=(Mesoinositol)) OR TS=(Myoinositol)) OR TS=(Fish Oils)) OR TS=(Liver Oils, Fish)) OR TS=(Vitamin D)) OR TS=(Minerals)) OR TS=(Magnesium)) OR TS=(Probiotics)) OR TS=(Antioxidants)) OR TS=(Zinc)) OR TS=(Vitamin B12)) OR TS=(Cyanocobalamin)) OR TS=(Folic Acid)) OR TS=(Pteroylglutamic Acid)) OR TS=(Vitamin B9)) OR TS=(Vitamin M)) OR TS=(Coenzyme Q10)) OR TS=(Ubiquinone)) OR TS=(Coenzyme Q)) OR TS=(Vitamin A)) OR TS=(Vitamin E)) OR TS=(Carnitine)) OR TS=(Levocarnitine)) OR TS=(Fatty Acids, Omega-3)) OR TS=(N-3 Fatty Acid)) OR TS=(Selenium)) OR TS=(Selenium-80)) OR TS=(Resveratrol)) OR TS=(Curcumin)) OR TS=(Curcumin Phytosome)) OR TS=(nutritional supplements)) OR TS=(Dietary Supplements)

#2 (TS=(Polycystic Ovary Syndrome)) OR TS=(PCOS)

#3 #1 AND #2 (2,345)
