# Supplementary material for: Efficacy of dietary supplements as an adjunctive therapy for polycystic ovary syndrome: an umbrella meta-analysis
Source: Front Nutr. 2025 Oct 29;12:1705284. doi: 10.3389/fnut.2025.1705284 (PMC12605168; doi:10.3389/fnut.2025.1705284)
Supplement: Supplementary file 3 [file Table_3.docx]

**Characteristics of meta-analyses examining the effects of nutritional supplements on PCOS**

| Study | Year | Country | Patients | Therapy | | Outcome | Quality |
| --- | --- | --- | --- | --- | --- | --- | --- |
|  |  |  |  | Experimental Group | Control Group |  |  |
| Arentz, S. | 2017 | Australia | 1406 | 1-4000 mg per day EPA 675 mg; DHA 1400 mg;2-4000 mg omega 3 (EPA 720 mg; DHA 480 mg);3-3500 mg (EPA: 2450 mg; DHA: 1210 mg) | 1-Placebo Olive oil 4000 mg;2-Placebo liquid paraffin 2000 mg in four capsules;3-Placebo (soybean oil 3500 mg) | total cholesterol;fasting glucose;HOMA-IR;LDL | Yes(Random;Allocation;)No(Blinding;Incomplete;Selective;)Low |
| Hajishafiee, M. | 2016 | Iran | 298 | 1-n-3 PUFA supplementation 4g/d:56%DHA, 27% EPA;2-Replacement of 31g/1800kcal of dietary fat with 36g walnut=31g oil: 2.9g SFA, 4.5g MUFA, 19.2g LA,4.3g ALA;3-Replacement of dietary fat with PUFA (walnut 48g/800kcal). 48g of walnut: 311kcal, 19g LA,3.3g ALA;4-n-3 PUFA supplementation 2.3g/d (2.1g EPA+DHA);5-n-3 PUFA supplementation 3g/d;6-n-3 PUFA supplementation 1500mg w-3/d;7-n-3 PUFA supplementation 4g/d: 2.4g n-3 PUFA, 1.9g EPA & DHA EPA/DHA:1.49/1;8-n-3 PUFA supplementation 3.5g/d;fish oil: 6 capsules/d (each capsule: 358mg EPA, 242mg DHA);flaxseed oil: 6 capsules/d (each capsule: 545mg w-3) | 1-Placebo(olive oil 4g/d);2-Replacement of 31g/1800kcal of dietary fat with 46g almond=31g oil;3-Placebo(paraffin);4-Placebo(olive oil 4g/d);5-Placebo(soybean oil 6 capsules/d) | total testosterone;SHBG levels | Yes(Random;Blinding;Incomplete;)No(Allocation;Selective;)Low |
| Sadeghi, A. | 2017 | Iran | 145 | 1-EPA/DHA dose 1.2 g;2-EPA/DHA dose 3.32 g;3-EPA/DHA dose 3.6 g | 1-Placebo;2-Placebo;3-Placebo | insulin resistance;HOMA –IR | Yes(Random;Blinding;Allocation;)No(Incomplete;Selective;)Low |
| Tosatti, Jessica A. G. | 2021 | Brazil | 384 | 1-2000 mg/d of fish oil per d;2-2000 mg/d of fish oil plus 50 000 IU of vitamin D every 2 weeks;3-180 mg of EPA and 120 mg of DHA per d;4-1000 mg/d of flaxseed oil;5-720 mg EPA and 480 mg DHA per d;6-540 mg EPA and 360 mg DHA per d;7-720 mg EPA and 480 mg DHA per d;8-400 mg of ALA plus 400 IU of vitamin E per d;9-400 mg of ALA plus 400 IU of vitamin E per d;10-545 mg ALA per d;11-358 mg EPA plus 242 mg DHA per d | 1-100 mg of paraffin oil per d;2-Not reported;3-1000 mg of paraffin oil per d;4-500 mg of paraffin oil per d;5-Four capsule contained 500 mg paraffin oil;6-1000 mg of paraffin oil per d;7-Paraffin oil;8-Not reported;9-Paraffin oil;10-Soya oil;11-Soya oil | adiponectin concentrations;visfatin concentrations;NO;GSH;MDA;TAC | Yes(Blinding;Incomplete;Selective;)No(Random;Allocation;)Low |
| Xia, Y. | 2021 | China | 778 | 1-[dosage (mg/d)] Fish oil (2,000);EPA (mg) 240;DHA (mg) 160;2-[dosage (mg/d)] Flaxseed oil (1,000) + VE (400 IU/d);EPA (mg) NA;DHA (mg) NA;3-[dosage (mg/d)] Fish oil (3,500);EPA (mg) 358;DHA (mg) 242;4-[dosage (mg/d)] Flaxseed oil (3,500);EPA (mg) 358;DHA (mg) 242;5-[dosage (mg/d)] Omega-3 supplement (2,000);EPA (mg) 180;DHA (mg) 120;6-[dosage (mg/d)] Omega-3 supplement (1,000);EPA (mg) 180;DHA (mg) 120;7-[dosage (mg/d)] Flaxseed oil (1,000);EPA (mg) NA;DHA (mg) NA;8-[dosage (mg/d)] Omega-3 supplement (4,000);EPA (mg) 180;DHA (mg) 120;9-[dosage (mg/d)] Omega-3 supplement (4,000);EPA (mg) 180;DHA (mg) 120;10-[dosage (mg/d)] Flaxseed oil (1,000) + VE (400 U/d);EPA (mg) NA;DHA (mg) NA;11-[dosage (mg/d)] Flaxseed oil (1,000) + VE (400 U/d);EPA (mg) NA;DHA (mg) NA | 1-Placebo;2-Placebo;3-Soybean oil;4-Soybean oil;5-Olive oil;6-Placebo;7-Placebo;8-Placebo;9-Placebo;10-Placebo;11-Placebo | insulin;HOMA-IR;total cholesterol (TC);triglyceride;LDL-C;VLDL-C;hs-CRP;HDL-C;serum glucose | Yes(Random;Blinding;Incomplete;Selective;)No(Allocation;)Moderate |
| Yang, K. | 2018 | China | 591 | 1-Omega-3 fatty acids 4000 mg;2-Omega-3 fatty acids (including fish oils and flaxseed oils);3-Omega-3 fatty acids 900 mg;4-Omega-3 fatty acids 900 mg;5-Omega-3 fatty acids 1000 mg + Vitamin E 400 IU + Metformin;6-Omega-3 fatty acids 1000 mg + vitamin E 400 IU;7-Omega-3 fatty acids 2000 mg;8-Omega-3 fatty acids 2000 mg + Metformin 500 mg;9-Omega-3 fatty acids 1000 mg + Vitamin E 400 IU | 1-Paraffin oil (placebo) 2000 mg;2-Soybean oil (placebo);3-Paraffin oil (placebo) 3000 mg;4-Paraffin oil (placebo) 3000 mg;5-Placebos + Metformin;6-Placebos;7-Olive oil (pla;8-cebo) 2000 mg;8-Paraffin oil (placebo) 1000 mg + Metformin 500 mg;9-Paraffin oil (placebo) | HOMA;total cholesterol;TG;Adiponectin;BMI;hyperinsulinemia;fasting glucose;LDL-C;HDL-C;FSH;LH;SHGB;total testosterone | Yes(Random;Allocation;Blinding;Incomplete;Selective;)High |
| Yuan, J. | 2021 | China | 610 | 1-Fish oil [2,000 mg/d];2-Flaxseed oil [1,000 mg/d] + VE (400 IU/d);3-Multinutrient [500 mg/d];4-Fish oil [2,000 mg/d] +VD (357 IU/d);5-Fish oil [3,500 mg/d];6-Flaxseed oil [3,500 mg/d];7-Flaxseed oil [1,000 mg/d];8-mega-3 supplement [3,000 mg/d];9-Flaxseed oil [1,000 mg/d] + VE (400 U/d);10-flaxseed oil [1,000 mg/d] + VE (400 IU/d);11-Fish oil [3,500 mg/d];12-Flaxseed oil [3,500 mg/d] | 1-Placebo;2-Placebo;3-Placebo;4-Placebo;5-Soybean oil;6-Soybean oil;7-Placebo;8-Placebo;9-Placebo;10-Placebo;11-Soybean oil;12-Soybean oil | CRP;GSH;MDA;TAC;DHEAS;FAI;FSH;LH;SHBG;TT | Yes(Random;Blinding;Incomplete;Selective;)No(Allocation;)Moderate |
| Zhou, J. | 2023 | China | 816 | 1-Fish oil n-3 PUFA sup- plements 2000 mg/d (Marine derived);2-Fish oil n-3 PUFA 4000 mg/d (Marine derived);3-Flaxseed oil n-3 PUFA 1000 mg/d (Plant origins)+vitamin E 400 IU/d;4-Fish oil n-3 PUFA 2000 mg/d (Marine derived)+vitamin E 50,000 IU/2 weeks;5-n-3 PUFA supple- ments 2000 mg/d (360 mg EPA and 240 mg DHA) (Marine derived);6-n-3 PUFA supple- ments 1000 mg/d (180 mg EPA and 120 mg DHA);7-Flaxseed oil n-3 PUFA 2000 mg/d (Plant origins);8-n-3 PUFA supple- ments 4000 mg/d (720 mg EPA and 480 mg DHA) (Marine derived);9-n-3 PUFA supple- ments 1000 mg/d (180 mg EPA and 120 mg DHA);10-Flaxseed oil n-3 PUFA 1000 mg/d (Plant origins)+Vita- min E 400 IU/d | 1-Parafn oil (placebo) 100 mg/d;2-olive oil (placebo) 4000 mg/d;3-Placebo;4-Placebo;5-Olive oil (placebo) 2000 mg/d;6-Parafn oil (placebo) 1000 mg/d;7-Parafn oil (placebo) 500 mg/d;8-Parafn oil (placebo) 500 mg/d;9-Parafn oil (placebo) 1000 mg/d;10-Placebo | BW;BMI;WC levels;FPG levels;FINS;HOMA-IR;QUICKI;Adiponectin;TG;TC;HDL-C;LDL-C;hs-CRP; | Yes(Random;Allocation;Blinding;Incomplete;Selective;)High |
| Abu-Zaid, A. | 2024 | Saudi Arabia | 345 | 1-Alpha-Lipoic Acid | 1-Placebo | BMI;FBS;HOMA-IR;insulin levels;estrogen;FSH;LH;testosterone | Yes(Random;Blinding;Selective;)No(Allocation;Incomplete;)Low |
| Fadlalmola, Hammad Ali | 2023 | Saudi Arabia | 218 | 1-Resveratrol 400 mg/twice daily, orally;2-Resveratrol 1500 mg/day, orally;3-Resveratrol 800 mg/day, orally;4-Resveratrol 1000  mg/day, orally | 1-placebo;2-placebo;3-placebo;4-placebo | Total testosterone;FSH;LH;Prolactin;TSH;DHEAS;pregnancy rates;Cholestero;HDL-C;LDL-C;Triglycerides;Acne score;C-reactive protein (CRP);Insulin;Sex hormone binding globulin (SHBG) | Yes(Random;Blinding;Incomplete;Selective;)No(Allocation;)Moderate |
| Abdelazeem, B. | 2022 | USA | 296 | 1-Highly bioavailable gel optimized Curcumin 93.34 mg QD;2-Curcumin 500 mg TID;3-Curcumin 500 mg QD;4-Curcumin 500 mg BID;5-Curcumin nanomicelle/ Metformin 80 mg QD/500 mg TID | 1-Placebo;2-Placebo (maltodextrin);3-Placebo (starch);4-Placebo;5-Metformin | fasting blood glucose;insulin level;homeostasis model assessment of insulin resistance;quantitative insulin sensitivity check index;total cholesterol; | Yes(Allocation;Blinding;Incomplete;Selective;)No(Random;)Moderate |
| Chien, Y. J. | 2021 | China Taiwan | 168 | 1-Curcumin 500 mg BID;2-Curcumin 500 mg TID;3-Curcumin 500 mg QD | 1-Placebo BID;2-Placebo(maltodextrin) TID;3-Placebo (starch) QD |  | Yes(Random;Blinding;Incomplete;)No(Allocation;Selective;)Low |
| Luis E. Simental‑Mendía | 2022 | Mexico | 296 | 1-Curcumin 1 g/day;2-Curcumin 93.34 mg/day;3-Curcumin 500 mg/day;4-Curcumin 1.5 g/day;5-Curcumin 80 mg/day+metformin 1.5 g/day | 1-Placebo;2-Placebo;3-Placebo;4-Placebo;5-Metformin 1.5 g/day | fasting glucose;insulin levels;HOMA-IR index;TC;LDL-C;HDL-C;triglycerides; | Yes(Random;Incomplete;Selective;)No(Allocation;Blinding;)Low |
| Mehran Nouri | 2022 | Iran | 198 | 1-curcumin 500(mg/d);2-curcumin 500(mg/d);3-curcumin 93.34(mg/d);4-curcumin 1500(mg/d) | 1-placebo;2-placebo;3-placebo;4-placebo | FBS;insulin level;HOMA-IR;QUICKY;BMI;insulin level; | No(Random;Allocation;Blinding;Incomplete;Selective;)Low |
| Shen, W. | 2022 | China | 447 | 1-Curcumin 500mg, qd;2-Curcumin 80mg, qd;Metformin 500mg, tid;3-Curcumin 500mg, bid;4-Curcumin 93.34mg;5-Curcumin 500mg, tid;6-CL water decoction 45ml, bid;7-CL water decoction 45ml, bid;Metformin 0.85g, bid | 1-Placebo Nr;2-Metformin 500mg, tid;3-Placebo Nr;4-Placebo Nr;5-Placebo 500mg, tid;6-Placebo 45ml,bid;7-Metformin 0.85g, bid | weight;waist circumference (WC);BMI;WHR;CRP;FBG;INS;QUICKI;HOMA-IR;2h OGTT;Ins120;glycosylated hemoglobin A1c (HbA1c);TC;TG;LDL-C;HDL-C;testosterone;dehydroepiandrosterone-sulfate (DHEA);LH;FSH;FAI;level of RBC;WBC;Cr;ALT;AST;adverse events; | Yes(Random;Incomplete;Selective;)No(Allocation;Blinding;)Low |
| Xie, Liangzhen | 2019 | China | 567 | 1-curcumin 500(mg/d);2-curcumin 500(mg/d);3-curcumin 93.34(mg/d);4-curcumin 1500(mg/d) | 1-placebo;2-placebo;3-placebo;4-placebo | pregnancy;total testosterone;SHBG;FAI;LH;fasting plasma glucose;insulin levels;postprandial plasma glucose level;HOMA-IR;total cholesterol;triglycerides;LDL-C;HDL-C;BMI;WC;WHR;gastrointestinal adverse events;events during pregnancy;live birth rate;conception;ovulation per subject;ovulation per cycle;Serious events during pregnancy | Yes(Random;)No(Allocation;Blinding;Incomplete;Selective;)Low |
| Caroline Cristine Almeida Balieiro | 2024 | Brazil | 916 | 1-Green tea;2-isoflavones(50 mg);3-Quercetin(1000 mg);4-Green tea;5-Quercetin(1000 mg);6-Quercetin(500 mg) | 1-Placebo;2-Placebo;3-Placebo;4-Placebo;5-Placebo;6-Placebo | insulin level;BMI;LH levels;testosterone levels; | Yes(Random;Blinding;Incomplete;Selective;)No(Allocation;)Moderate |
| Lesani, A. | 2022 | Iran | 139 | 1-540 mg/d Green tea extract capsule;2-500 mg/d Green tea capsule;3-500 mg/d Green tea tablet | 1-Placebo;2-Placebo;3-Placebo | weight;fasting insulin;waist to hip ratio;body mass index;body fat percentage | Yes(Random;Allocation;Blinding;Incomplete;Selective;)High |
| Heshmati, Javad | 2018 | Iran | 268 | 1-Chromium picolinate 200μg daily;2-Chromium picolinate 1000μg daily;3-Chromium picolinate 500mg twice daily;4-Chromium 200μg;5-Chromium picolinate 200μg | 1-Metformin;2-placebo;3-MET 500mg thrice daily;4-Placebo;5-Placebo | fasting insulin;QUICKI;HOMA-IR;FSI; | Yes(Random;Allocation;Blinding;Incomplete;Selective;)High |
| Siavash, Fazelian | 2017 | Iran | 351 | 1-200 mcg/day Cr picolinate;2-1000 mcg/day Cr picolinate;3-1000 mcg/day Cr picolinate;4-200 mcg/day Cr picolinate;5-200 mcg/day Cr picolinate;6-1000 mcg/day Cr picolinate;7-200 mcg/day Cr picolinate | 1-Placebo;2-nr;3-nr;4-Placebo(cellulose);5-Placebo(cellulose);6-placebo;7-1500 mg/day Metformin | FBS;fasting insulin;BMI;free testosterone;total testosterone;FG;DHEA;FSH;LH; | Yes(Random;Blinding;Incomplete;)No(Allocation;Selective;)Low |
| Ahmed Abu-Zaid | 2023 | Saudi Arabia | 413 | 1-Selenium dosage 200(μg/day);2-Selenium dosage 200(μg/day);3-Selenium dosage 200(μg/day);4-Selenium dosage 200(μg/day);5-Selenium dosage 200(μg/day);6-Selenium dosage 200(μg/day);7-Selenium dosage 200(μg/day) | 1-Placebo;2-Placebo;3-Placebo;4-Placebo;5-Placebo;6-Placebo;7-Placebo | FPG;insulin;HOMA-IR;QUICKI;TC;TG;HDL;LDL;VLDL;MDA;hs-CRP;TAC;GSH;NO;SHBG;total testosterone;MFG score;total testosterone; | Yes(Random;Allocation;Blinding;)No(Incomplete;Selective;)Low |
| Arentz, S. | 2017 | Australia | 1406 | 1-Selenium 200mcg per day;2-Selenium 200mcg plus Metformin 1500 mg per day; | 1-Placebo (type not specified);2-Placebo (cellulose) plus Metformin 1500 mg per day | fasting glucose;HOMA-IR;total testosterone;SHBG;FAI | Yes(Random;Allocation;)No(Blinding;Incomplete;Selective;)Low |
| Pei-yu Wu | 2022 | China | 317 | 1-200 μ g/day selenium for 12 weeks;2-200-μg selenium daily for 12 weeks;3-200 µg/day selenium for 12 weeks;4-200 µg/day selenium for 12 weeks;5-200 µg/day selenium for 8 weeks | 1-placebo;2-placebo;3-placebo;4-placebo;5-placebo | total testosterone;SHBG;cholesterol;triglyceride;LDL;FPG;HOMA-IR; | Yes(Random;Blinding;Incomplete;Selective;)No(Allocation;)Moderate |
| Ziaei, S. | 2024 | Iran | 1006 | 1-Melatonin dosage 6(mg/day);2-Melatonin dosage 10(mg/day);3-Melatonin dosage 10(mg/day);4-Melatonin dosage 6(mg/day);5-Melatonin dosage 3(mg/day);6-Melatonin dosage 3(mg/day) | 1-Placebo;2-Placebo;3-Placebo;4-Placebo;5-Placebo;6-Placebo | weight;BMI;TAC levels;FBS;Insulin;HOMA-IR;TC;TG;HDL;LDL;MDA;hs-CRP;mFG;SHGB;Total Testosterone;Endometrial thickness;Pregnancy Rate | Yes(Random;Allocation;Blinding;)No(Incomplete;Selective;)Low |
| Arentz, S. | 2017 | Australia | 1406 | 1-Inositol 2 g and folic acid 200 mg plus folic acid 200mcg;2-Inositol 4 g + folic acid 400mcg;3-Inositol 1200 mg/day as powder,pre-dosed presented in sachets dissolved in water;4-Inositol 200 mg;5-Inositol 4 g plus folic acid 400mcg;6-Inositol (chiro) 600 mg;7-Inositol 4 g + folic acid 400mcg | 1-Folic acid 400mcg daily;2-Folic acid 400mcg;3-Placebo;4-Placebo matched to Gestosan;5-Folic acid 400mcg;6-Placebo;7-Metformin | Free testosterone;Total testosterone;Androstenidione;Sex hormone binding globulin (SHBG);Modified Ferriman Gallwey score;Number of days to ovulation;Number of ovulations;Pregnancy;Live births;FSH;LH;FSH:LH ratio;BMI;Waist to hip ratio;Fasting glucose;Fasting insulin;HOMA-IR;Cholesterol;Triglycerides; | Yes(Random;Allocation;)No(Blinding;Incomplete;Selective;)Low |
| Greff, D. | 2023 | Hungary | 1691 | 1-MI 1000 mg 24w;2-MI (1100 mg)+DCI (27,6 mg) 24w;3-MI (2000 mg)+DCI (400 mg) 12w;4-MI 1000 mg 16w;5-MI 4000 mg 12w;6-MI 4000 mg 12-16w;7-MI 4000 mg 12-16w;8-1. MI 4000 mg 24w;2. MI 1100 mg+DCI 27,6 mg 24w;9-MI 4000 mg 24w;10-MI 2000 mg 12w;11-MI 4000 mg 14w;12-MI 4000 mg 12w;13-DCI 600 mg 7w;14-MI 3000 mg 24w;15-MI 4000 mg 12w;16-MI 2000 mg 24w;17-MI 2000 mg 24w;18-DCI 1200 mg 7w;19-MI 4000 mg 12w;20-MI 4000 mg 24w;21-MI 4000 mg 12w;22-MI 4000 mg NR w;23-MI 4000 mg 12w;24-MI 4000 mg 12w;25-MI 4000 mg 24w;26-MI 1000 mg 24w | 1-MET 1000 mg;2-FA 400mcg;3-FA 400mcg;4-MET 1000 mg;5-MET 1700 mg;6-FA 400 mcg;7-Placebo powder;8-diet;9-MET 1500 mg;10-FA 200mcg;11-FA 400mcg;12-MET 1500 mg;13-NR;14-MET 1700 mg;15-MET 1500 mg;16-MET 1500 mg;17-MET 1500 mg;18-Placebo;19-1.MET 1500 mg;2.FA 400mcg;20-MET 1500 mg;21-MET 1700 mg;22-FA 400mcg;23-MET 1500 mg;24-FA 500mcg;25-MET 1500 mg;26-MET 1700 mg | the rate of cycle normalization;BMI;weight;total testosterone levels;free testosterone;SHBG levels;Androstenedione;androstenedione;DHEAS;fasting plasma glucose;glucose levels;AUC-insulin levels;pregnancy rate;cycle normalization;SHBG;FG-score;pregnancy rate;Side efects; | Yes(Random;Blinding;)No(Allocation;Incomplete;Selective;)Low |
| Pundir, J. | 2017 | UK | 601 | 1-Inositol (Myo-inositol or di-chiro-inositol);2-Myo-inositol;3-Myo-inositol;4-Myo-inositol;5-Inositol (Myo-inositol or di-chiro-inositol) | 1-placebo;2-Di-chiro-inositolvs. placebo;3-Di-chiro-inositol;4-metformin;5-Clomiphene | ovulation rate;frequency of menstrual cycles;pregnancy;total androgen;total testosterone;free testosterone;serum dehydroepiandrosterone;sex-hormone-binding globulin;fasting insulin;fasting glucose;HOMA-IR; | Yes(Random;Blinding;Incomplete;Selective;)No(Allocation;)Moderate |
| Unfer, Vittorio | 2017 | Switzerland | 486 | 1-MI+FA;2-MI+FA;3-MI+FA;4-MI+FA;5-MI+FA;6-MI+FA;7-MI+FA;8-MI+DCI;9-MI+DCI | 1-FA;2-FA;3-FA;4-FA;5-DCI+FA;6-OCPs;7-COC;8-MI;9-FA | fasting insulin;HOMA-IR;serum testosterone;androstenedione;SHBG levels; | Yes(Incomplete;Selective;)No(Random;Allocation;Blinding;)Low |
| Zeng, L. | 2018 | China | 573 | 1-MI 0.55 g, DCI 0.0138 g and folic acid 200 μg b.i.d;2-MI 1 g b.i.d;3-MI 4 g and folic acid 400 μg q.d.;4-MI 2 g and folic acid 200 μg q.d.;5-MI 1.2 g q.d.;6-MI 4 g and folic acid 400 μg q.d.;7-MI 2 g and folic acid 200 μg q.d.;8-Group I: MI 2 g and folic acid 200 μg; Group III MI 2 g, folic acid 200 μg and drospirenone 3 mg/ethinyl estradiol 30 μg;9-MI 2 g and folic acid 200 μg b.i.d;10-MI 0.1 g b.i.d | 1-Folic acid 200 μg b.i.d;2-Metformin 500 mg t.i.d.;3-Metformin 1.5 g q.d.;4-Folic acid 400 μg q.d.;5-Placebo 1.2 g q.d.;6-Folic acid 400 μg q.d.;7-Folic acid 200 μg q.d.;8-Group II:Drospirenone 3 mg/ethinyl estradiol 30μg;9-Folic acid 200 μg b.i.d;10-Placebo 0.1 mg b.i.d | HOMA-IR;total testosterone;fasting glucose;free testosterone;SHBG;androstenedione;FSH;LH; DHEAS | Yes(Random;Blinding;Incomplete;)No(Allocation;Selective;)Low |
| Hajar Heidari | 2022 | Iran | 504 | 1-1000 mg omega-3 fatty acids plus 400 IU vitamin E supplements；2-1000 mg omega-3 fatty acids from faxseed oil  containing 400 mg α-linolenic acid plus 400 IU vitamin E supplements；3-1000 mg omega-3 fatty acids from faxseed oil containing 400 mg α-Linolenic acid plus 400 IU vitamin E supplements；4-400 IU vitamin E；5-250 mg/day magnesium plus 400 mg/ day vitamin E supplements；6-400 IU vitamin E；7-1000 mg omega-3 plus 400 IU vitamin E supplements；8-250 mg/day magnesium plus 400 mg/day vitamin E supplements；9-2 g of omega-3 plus 400 IU of vitamin E；10-400 IU/day vitamin E -as alpha tocopheryl acetate | 1-Placebo (parafn)；2-Placebo；3-Placebo；4-Placebo；5-Placebo；6-Placebo；7-Placebo；8-Placebo；9-Placebo；10-Placebo | TG;VLDL;LDL-c;TC;TC /HDL-c ratio;TAC;GSH;MDA;hs-CRP;NO levels;FBS;insulin;HOMA-IR;QUICKI;total testosterone;LH;FSH;SHBG;FAI;weight;BMI;waist circumference;hirsutism score | Yes(Random;Blinding;Incomplete;Selective;)No(Allocation;)Moderate |
| Sebastián Yalle-Vásquez | 2022 | Peru | 540 | 1-Vitamin E 400 IU þ Omega-3 1000 mg daily for 3 months;2-Vitamin E 400 mg þ 50 Vitamin D3000 IU/L twice a week or 3 300 IU/day for 10 weeks;3-Vitamin E 400 IU daily for 2 months;4-Vitamin E 400 IU þ Omega-3 100 mg daily for 3 months;5-Vitamin E 400 mg þ Magnesium oxide 250 mg daily for 3 months;6-Vitamin E 1500 IU daily for 3 menstrual cycles;7-Vitamin E 300 mg/dl 3 times daily for 2 months;8-Vitamin E 400 IU daily for 2 months | 1-placebo;2-placebo;3-placebo;4-placebo;5-placebo;6-placebo;7-placebo;8-placebo | fasting serum glucose;HOMA-IR;fasting serum insulin;BMI;testosterone;free androgen index;SHBG;Ferriman-Gallwey score;total cholesterol;LDL-cholesterol;triglyceride levels;HDL-cholesterol levels | Yes(Random;Incomplete;Selective;)No(Allocation;Blinding;)Low |
| Akbari, M. | 2018 | Iran | 406 | 1-1000 IU vitamin D/day;2-50000 IU vitamin D/every 2 weeks;3-1000 IU vitamin D/day + 1 g EPO/day;4-400 IU vitamin D/day + other nutrients/day;5-400 IU Vitamin D/day + 180 μg vitamin K/day + 1 g calcium carbonate/day;6-50000 IU vitamin D/week + 1 g calcium carbonate/day;7-50000 IU vitamin D/every 20 days | 1-placebo;2-placebo;3-placebo;4-placebo;5-placebo;6-placebo;7-placebo | hs-CRP;NO;TAC levels;GSH;MDA | Yes(Random;Allocation;Blinding;Selective;)No(Incomplete;)Moderate |
| Fang, F. 2017 | 2017 | China | 502 | 1-Vitamin D 50000 IU/20 days;2-Vitamin D 50000 IU/20 days;3-Vitamin D 50000 IU/week + Ca 1000 mg/day;Vitamin D 50000 IU/week + Ca placebo;4-Vitamin D 0.5 mg/day;5-Vitamin D 50000 IU/week + Ca 1000 mg/day + merformin 1500 mg/day;6-Vitamin D 4000 IU/day + metformin 1500 mg/day;7-Vitamin D 12000 IU/day;8-Vitamin D 400 IU/day + Ca 1000 mg/day + metformin 1500 mg/day;Vitamin D 400 IU/day + Ca 1000 mg/day;9-Vitamin D 50,000 IU/2 weeks + Ca 1000 mg/day + metformin 1500 mg/day;Vitamin D 50000 IU/2 weeks + Ca 1000 mg/day | 1-Placebo;2-Placebo;3-Vitamin D placebo + Ca 1000 mg/day;Vitamin D placebo + Ca placebo;4-Metformin 1000 mg/day;Placebo;5-Metformin 1500 mg/day;6-Metformin 1500 mg/day + placebo;Placebo;7-Metformin 1500 mg/day;8-Metformin 1500 mg/day;9-Placebo | serum 25(OH)D;Serum PTH levels;the number of dominant follicles;regular menstrual cycles;follicle development | Yes(Random;Allocation;Blinding;Incomplete;Selective;)High |
| Gao, H. | 2021 | China | 543 | 1-Vitamin D | 1-Placebo | total cholesterol level;triglyceride level;LDL-C; | Yes(Random;Allocation;Selective;)No(Blinding;Incomplete;)Low |
| Han, Y. | 2023 | China | 849 | 1-Vitamin D + calcium;2-Vitamin D;3-Vitamin D + calcium + vitamin K2;4-Metformin + vitamin D;5-Metformin + calcium + vitamin D;6-Vitamin D;7-Vitamin D;vitamin D + metformin;8-Metformin + calcium + vitamin D;9-Vitamin D;10-Vitamin D + calcium;Metformin + calcium + vitamin D;11-Metformin + calcium + vitamin D;Vitamin D + calcium;12-Vitamin D | 1-Calcium;2-Placebo;3-Placebo;4-Metformin;5-Metformin;6-Placebo;7-Metform;8-Metformin;9-Placebo;10-Metformin;11-Placebo;Metformin;12-Placebo | LH;FSH;LH/FSH;menstrual cycle; | Yes(Random;Incomplete;Selective;)No(Allocation;Blinding;)Low |
| Jin, B. | 2020 | China | 467 | 1-Cholecalciferol;2-Cholecalciferol;3-Cholecalciferol;4-Cholecalciferol + Calcium;5-Cholecalciferol + MET;6-Cholecalciferol;7-Cholecalciferol(high-dose) + MET;Cholecalciferol(lose-dose) + MET;8-Cholecalciferol;9-Cholecalciferol | 1-Placebo;2-Placebo;3-Placebo;4-Placebo + Calcium;5-Placebo + MET;6-Placebo;7-Placebo + MET;8-Placebo;9-Placebo | triglyceride levels;total cholesterol;low-density lipoprotein cholesterol;very-low-density lipoprotein cholesterol;high-density lipoprotein cholesterol levels | Yes(Random;Blinding;Incomplete;Selective;)No(Allocation;)Moderate |
| Luo, J. | 2021 | China | 677 | 1-Vitamin D;2-Vitamin D;Vitamin D + calcium;3-Vitamin D;4-Low-dose vitamin D;High-dose vitamin D;5-Vitamin D + metformin;6-Vitamin D;7-Vitamins D and K + calcium;8-Vitamin D;9-Vitamin D + EPO;10-Vitamin D;11-Vitamin D | 1-Placebo;2-Placebo;3-Placebo;4-Placebo;5-Placebo + metformin;6-Placebo;7-Placebo;8-Placebo;9-Placebo;10-Placebo;11-Placebo | TC;TG;LDL-C;HDL;VLDL-C | Yes(Random;Allocation;Blinding;Selective;)No(Incomplete;)Moderate |
| Miao, C. Y. | 2019 | China | 483 | 1-Weight-loss intervention + 50,000 IU/week oral vitamin D3;2-50,000 IU of oral vitamin D3;3-50,000 IU vitamin D every 2 weeks;4-50,000 IU of vitamin D3 every 20 days;5-Vitamin D3 12,000 IU daily;6-Metformin (1,500 mg/day) + vitamin D 4,000 IU/day (monthly dose of 120,000 IU);7-50,000 IU vitamin D every other week;8-Vitamin D 3200 IU daily;9-50,000 IU of oral vitamin D3 once weekly;10-50,000 IU vitamin D weekly and calcium placebo daily;11-Calcitriol 0.5 µg/day | 1-Weight loss intervention + placebo;2-Placebo;3-Placebo;4-Placebo;5-Placebo;6-Metformin (1,500 mg/day) + placebo;7-Placebo;8-Placebo;9-Placebo once weekly;10-Calcium placebo daily plus vitamin D placebo weekly;11-Placebo | BMI;total testosterone;DHEA;HOMA-IR;total cholesterol;LDL-C;triglyceride;HDL-C | Yes(Random;Allocation;)No(Blinding;Incomplete;Selective;)Low |
| Pergialiotis, V. | 2017 | Greece | 647 | 1-Ca+vD;Ca+vD+Met;2-Met;vD;3-Vitamin D;4-Met;Met+Ca+vD;Ca+vD;5-vD3;6-vD+Met;7-vD;8-vD+vK+Ca;9-vD;vD+Met | 1-Met;2-placebo;3-placebo;4-placebo;5-placebo;6-placebo;7-placebo;8-placebo;9-Met | testosterone;LH;DHEAS;fasting glucose;fasting insulin;HOMA-IR; | Yes(Random;Allocation;Incomplete;Selective;)No(Blinding;)Moderate |
| Xue, Y. | 2017 | China | 855 | 1-nr;2-Vitamin D: 50,000 IU/wk 8 weeks;3-Vitamin D3: 50,000 IU/20 days 2 months;4-Vitamin D:400 IU orally 3 months;5-Vitamin D3:10,0000 IU/month 6 months;6-Vitamin D: 3,300,000 IU/day as a single oral dose 3 weeks;7-Cholecalciferol:300,000 IU,by intragluteal injection;8-Vitamin D3:4000 IU/day 6 months;9-Calcitriol:0.5 lg/day 3 month;10-Vitamin D3: 2000 IU/day + Vitamin D2 50,000 IU/month;11-Vitamin D3: 12000 IU/12 weeks 12 weeks;12-Vitamin D3: 20,000 IU/week 12 weeks;13-Vitamin D3: 50,000 IU/ 2 weeks 4 months;14-Ergocalciferol 50 000 IU weekly or biweekly;15-Vitamin D3: 50 000 IU oral 8 weeks;16-Vitamin D: 200 IU 8 weeks | 1-Placebo;2-Placebo;3-Placebo;4-Placebo;5-Placebo;6-Placebo;7-Placebo;8-Placebo;9-Placebo;10-Placebo;11-Placebo;12-Placebo;13-Placebo;14-Placebo;15-Placebo;16-Placebo | 25-hydroxy-vitamin D;HOMA-IR;QUICKI;serum triglyceride;LDL;PTH;DHEAS;FT;TT | Yes(Random;Allocation;Blinding;Incomplete;Selective;)High |
| Zhang, B. | 2023 | China | 840 | 1-VD3 300,000 IU;2-VD 4000 IU/day;3-VD 50,000 IU/week;4-VD 4000 or 1 000IU/day;5-VD 50,000 IU/2 weeks;6-VD 50,000 IU/2 weeks;7-200 IU VD/day;8-VD 50,000 IU/2 weeks;9-VD3 50,000 IU;10-VD3 12,000 IU/day;11-VD 200 IU/day;12-VD 200 IU/day;13-VD 400 IU/day | 1-Placebo;2-Placebo;3-Placebo;4-Placebo;5-Placebo;6-Placebo;7-Placebo;8-Placebo;9-Placebo;10-Placebo;11-Placebo;12-Placebo;13-Placebo | hs-CRP;Parathyroid hormone level;Total cholesterol level;Total testosterone level;(mF-G) score;Endometrial thickness | Yes(Random;Allocation;Incomplete;Selective;)No(Blinding;)Moderate |
| Li, Y. | 2023 | China | 1049 | 1-Probiotic;2-Probiotic;3-Synbiotic;4-Probiotic;5-Probiotic;6-Probiotic;7-Synbiotic;8-Synbiotic;9-Synbiotic;10-Probiotic;11-Synbiotic;12-Prebiotic;13-Probiotic;14-Prebiotic;15-Probiotic;16-Synbiotic;17-Probiotic | 1-Placebo (starch);2-Placebo;3-Placebo;4-Placebo(maltodextrin);5-Placebo;6-Placebo (starch);7-Placebo (starch and maltodextrin);8--Placebo (starch and maltodextrin);9-Placebo;10-Placebo (starch);11-Placebo (starch);12-Placebo (maltodextrin);13-Placebo;14-Placebo(maltodextrin);15-Placebo (starch and maltodextrin);16-Placebo;17-Placebo | FPG;FINS;HOMA-IR;QUICKI;TG;TC;HDL-c;LDL-c;VLDL-c;BW;BMI;WC;HC;CRP; | Yes(Random;Allocation;Incomplete;Selective;)No(Blinding;)Moderate |
| Miao, C. | 2021 | China | 486 | 1-Lactobacillus rhamnosus GG, Bacillus coagulans, Bacillus indicus;2-Lactobacillus acidophilus, Lactobacillus casei, Lactobacillus bulgaricus, Lactobacillus rham- nosus, Bifidobacterium longus, Bifidobacterium breve, Streptococcus thermophilus;3-Lactobacillus acidophilus, Lactobacillus casei, Bifidobacterium bifidum;4-Lactobacillus casei, Lactobacillus acidophilus, Lactobacillus rham- nosus Lactobacillus, Bulgaricus, Bifidobacterium breve, Bifidobacterium longum, Streptococcus thermophiles;5-20 g of prebiotic;6-Lactobacillus acidophilus, Lactobacillus casei, Lactobacillus bulgaricus, Lactobacillus rham- nosus, Bifidobacterium longum, Bifidobacterium breve, Streptococcus thermophilus;7-Bifid triple viable combined with metformin | 1-Unknown;2-Placebo capsules containing starch and maltodextrin, but no bacteria;3-Placebo;4-Placebo containing starch and maltodextrins but no bacteria;5-Placebo (maltodextrin);6-Placebo capsules containing starch and maltodextrin but no bacteria;7-Metformin | HOMA-IR;insulin;FBS;BMI;WC;HC | Yes(Random;Allocation;Incomplete;Selective;)No(Blinding;)Moderate |
| Reza, Tabrizi | 2022 | Iran | 730 | 1-Synbiotic capsule contained Lactobacillus acidophilus, Lactobacillus casei, and Bifidobacterium bifidum (2 × 109 CFU/g each) plus 800 mg inulin;2-Synbiotic capsule (500 mg) contained Lactobacillus acidophilus 3 × 1010 CFU/g, Lactobacillus casei 3 × 109 CFU/g, Lactobacillus bulgaricus 5 × 108 CFU/g, Lactobacillus rhamnosus 7 × 109 CFU/g, Bifidobacterium longum 1 ×109 CFU/g,Bifidobacterium breve 2 × 1010 CFU/g and Streptococcus thermophilus 3 × 108 CFU/g + prebiotic inulin(fructo-oligosaccharide);3-Probiotic capsule contained Lactobacillus acidophilus, Lactobacillus casei and Bifidobacterium bifidum (2 × 109 CFU/g each);4-Probiotic capsule (500 mg) contained Lactobacillus casei 7 × 109 CFU/g, Lactobacillus acidophilus 2 × 109 CFU/g,Lactobacillus rhamnosus 1.5 × 109 CFU/g, Lactobacillus bulgaricus 2 × 108 CFU/g, Bifidobacterium breve 2 × 1010 CFU/g,Bifidobacterium longum 7 × 109 CFU/g, Streptococcus thermophiles 1.5 × 109 CFU/g;5-Probiotic capsule contained Lactobacillus acidophilus, Lactobacillus casei and Bifidobacterium bifidum (2 × 109 CFU/g each);6-Probiotic capsule (1000 mg) contained Lactobacillus acidophilus,Lactobacillus plantarum, Lactobacillus fermentum, and Lactobacillus Gasseri (1 × 109 CFU of each);7-Synbiotic capsule contained Lactobacillus acidophilus, Lactobacillus casei, and Bifidobacterium bifidum (2 × 109 CFU/g each) plus 800 mg inulin;8-Probiotic capsule contained Lactobacillus acidophilus, Lactobacillus reuteri, Lactobacillus fermentum, and Bifidobacterium bifidum(2 × 109 CFU/g each) + 200 mcg selenium;9-Probiotic capsule contained Lactobacillus acidophilus, Lactobacillus reuteri, Lactobacillus fermentum, and Bifidobacterium bifidum(2 × 109 CFU/g each) + 200 mcg selenium;10-Synbiotic pomegranate juice 2 lit per week contained Lactobacillus(4 × 108 CFU/g) plus 40 g inulin;11-Synbiotic beverage 2 lit per week contained Lactobacillus(4 × 108 CFU/g) plus 40 g inulin;12-Probiotic capsule contained Lactobacillus acidophilus, Lactobacillus reuteri, Lactobacillus fermentum, and Bifidobacterium bifidum(2 × 109 CFU/g each) + 50,000 IU vitamin D every 2 weeks | 1-Placebo;2-Placebo;3-Placebo;4-Placebo;5-Placebo;6-Placebo;7-Placebo;8-Placebo;9-Placebo;10-Placebo;11-Placebo;12-Placebo | weight;BMI;FPG;insulin;HOMA-IR;triglycerides;VLDL-cholesterol;CRP;malondialdehyde (MDA);hirsutism;total testosterone levels;QUICKI;NO;total antioxidant capacity (TAC);glutathione (GSH);sex hormone binding globulin (SHBG) | Yes(Random;Allocation;Blinding;Selective;)No(Incomplete;)Moderate |
| Shamasbi, S. G. | 2020 | Iran | 855 | 1-receive either one synbiotics capsule per day for 12 weeks;2-intake either probiotic supplements for 12 weeks;3-receive either probiotic supplements for 12 weeks;4-two probiotic capsules per day for 12 weeks;5-probiotic supplement;probiotic group received one Familact probiotic capsule (500 mg);6-Participants in the intervention group received ten billions probiotic capsules twice daily, after lunch and evening meal for 12 weeks;7-200 µg/day selenium as selenium yeast plus 8000000000 CFU/day probiotic;8-active treatment with synbiotics;9-Prebiotic containing 20 g of resistant dextrin (polysaccharides produced  from maize, wheat and other edible starches);10-Synbiotics supplements were containing Lactobacillus acidophilus, Lactobacillus casei and Bifdobacterium bifdum plus 0.8 g inulin;11-intake either probiotic plus 200 μg/day selenium;12-1. Synbiotics pomegranate juice;2. Pomegranate juice;3. Synbiotics beverage;13-Prebiotic containing 20 g of resistant dextrin (polysaccharides produced from maize, wheat and other edible starches) | 1-placebo;2-placebo;3-placebo (starch);4-two maltodextrin capsules;5-placebo;6-placebo;7-placebo;8-placebo;9-placebo containing 20 g maltodextrin;10-placebo;11-placebo;12-2 L(2×1-l) of placebo beverage per 1 week, for 8 weeks in disposable bottles (each liter of beverage contains 1 l of water+2 pomegranate favoring);13-placebo containing 20 g maltodextrin | Testosterone;DHEAS;SHBG;FAI;hsCRP;NO;TAC;GSH;MDA;hirsutism | Yes(Allocation;)No(Random;Blinding;Incomplete;Selective;)Low |
| Tabrizi, R. | 2022 | Iran | 730 | 1-Synbiotic capsule contained Lactobacillus acidophilus, Lactobacillus casei, and Bifidobacterium bifidum plus 800 mg inulin;2-Synbiotic capsule (500 mg) contained Lactobacillus acidophilus, Lactobacillus casei, Lactobacillus bulgaricus, Lactobacillus rhamnosus, Bifidobacterium longum,Bifidobacterium breve,Streptococcus thermophilus+ prebiotic inulin(fructo-oligosaccharide);3-Probiotic capsule contained Lactobacillus acidophilus, Lactobacillus casei and Bifidobacterium bifidum;4-Probiotic capsule (500 mg) contained Lactobacillus casei, Lactobacillus acidophilus,Lactobacillus rhamnosus,Lactobacillus bulgaricus,Bifidobacterium breve,Bifidobacterium longum,Streptococcus thermophiles;5-Probiotic capsule contained Lactobacillus acidophilus, Lactobacillus casei and Bifidobacterium bifidum;6-Probiotic capsule (1000 mg) contained Lactobacillus acidophilus, Lactobacillus plantarum, Lactobacillus fermentum, and Lactobacillus Gasseri;7-Synbiotic capsule contained Lactobacillus acidophilus, Lactobacillus casei, and Bifidobacterium bifidum plus 800 mg inulin;8-Probiotic capsule contained Lactobacillus acidophilus, Lactobacillus reuteri, Lactobacillus fermentum, and Bifidobacterium bifidum + 200 mcg selenium;9-Probiotic capsule contained Lactobacillus acidophilus, Lactobacillus reuteri, Lactobacillus fermentum, and Bifidobacterium bifidum + 200 mcg selenium;10-Synbiotic pomegranate juice 2 lit per week contained Lactobacillus plus 40 g inulin;11-Synbiotic beverage 2 lit per week contained Lactobacillus plus 40 g inulin;12-Probiotic capsule contained Lactobacillus acidophilus, Lactobacillus reuteri, Lactobacillus fermentum, and Bifidobacterium bifidum + 50,000 IU vitamin D every 2 weeks | 1-placebo;2-placebo;3-placebo;4-placebo;5-placebo;6-placebo;7-placebo;8-placebo;9-placebo;10-placebo;11-placebo;12-placebo | weight;BMI;fasting plasma glucose (FPG);insulin;HOMA-IR;triglycerides;VLDL-cholesterol;CRP;malondialdehyde (MDA);hirsutism;total testosterone levels;QUICKI;NO;total antioxidant capacity (TAC);glutathione (GSH);sex hormone binding globulin (SHBG) levels | Yes(Random;Allocation;Blinding;Selective;)No(Incomplete;)Moderate |
| Gong, Y. | 2023 | China | 839 | 1-Metformin tablets at the initial dose of 500 mg and gradually increased to not more than 1500 mg daily;Carnitine, 250 mg daily;2-Carnitine, 250 mg daily;3-150 mg/day CC for 5 days plus oral and metformin 850 mg twice daily;L‐carnitine, 3000 mg daily;4-Metformin tablets at the initial dose of 500 mg and gradually increased up to 1500 mg daily;Carnitine, 250 mg daily;5-Combined metformin and pioglitazone (500/15 twice daily);Carnitine, 3000 mg daily;6-CC (250 mg per day) from Day 3 to 7 days of the cycle;Carnitine, 3000 mg daily;7-CC;Carnitine, 1000 mg daily | 1-Metformin tablets at the initial dose of 500 mg and gradually increased to not more than 1500 mg daily;Placebo;2-Placebo;3-150 mg/day CC for 5 days plus oral and metformin 850 mg twice daily;Placebo;4-Metformin tablets at the initial dose of 500 mg and gradually increased up to 1500 mg daily;Placebo;5-Combined metformin with pioglitazone (500/15 twice daily);Placebo;6-CC (250 mg per day) from Day 3 to 7 days of the cycle;Placebo;7-CC;Placebo | ovulation rates;pregnancy rates;insulin;HOMA-IR;BMI; | Yes(Random;Blinding;Selective;)No(Allocation;Incomplete;)Low |
| Shukri, Mohd Falihin Mohd | 2022 | Malaysia | 995 | 1-150 mg/day CC plus oral LC 3g and metformin 850 mg (1 tablet daily);2-150 mg/day of CC plus 3 g of oral LC daily, and placebo sachets;3-250 mg CC from day three until day seven of the cycle plus LC 3 g daily;4-250 mg carnitine supplements;5-LC 1,000 mg/d plus 200 mg/d chromium as chromium picolinate;6-200 µg/day chromium pi- colinate plus 1,000 mg/day LC;7-250 mg LC (capsule range 237–275 mg);8-250 mg/day of LC;9-Oral CC (50 mg tablet, two times per day) plus oral LC supplementation (1 g tablet, three times per day) | 1-150 mg/d CC plus metformin and placebo capsules;2-150 mg/day of CC from day 3 until day 7 of the menstrual cycle plus 600 mg of oral N-acetylcysteine three times daily, and a placebo capsule;3-250 mg CC with placebo;4-Placebos (cellulose);5-Placebo;6-Placebo (starch);7-Placebo (cellulose);8-Placebo;9-Oral CC only (50 mg tablet, two times per day) | fasting plasma glucose (FPG);LDL;total cholesterol;triglyceride(TG); | Yes(Random;Allocation;Blinding;Incomplete;Selective;)High |
| Arentz, S. | 2017 | Australia | 1406 | 1-4000 mg per day EPA 675 mg; DHA 1400 mg;2-4000 mg omega 3 (EPA 720 mg; DHA 480 mg);3-3500 mg (EPA: 2450 mg; DHA: 1210 mg) | 1-Placebo Olive oil 4000 mg;2-Placebo liquid paraffin 2000 mg in four capsules;3-Placebo (soybean oil 3500 mg) | total cholesterol;fasting glucose;HOMA-IR;LDL | Yes(Random;Allocation;)No(Blinding;Incomplete;Selective;)Low |
| Hajishafiee, M. | 2016 | Iran | 298 | 1-n-3 PUFA supplementation 4g/d:56%DHA, 27% EPA;2-Replacement of 31g/1800kcal of dietary fat with 36g walnut=31g oil: 2.9g SFA, 4.5g MUFA, 19.2g LA,4.3g ALA;3-Replacement of dietary fat with PUFA (walnut 48g/800kcal). 48g of walnut: 311kcal, 19g LA,3.3g ALA;4-n-3 PUFA supplementation 2.3g/d (2.1g EPA+DHA);5-n-3 PUFA supplementation 3g/d;6-n-3 PUFA supplementation 1500mg w-3/d;7-n-3 PUFA supplementation 4g/d: 2.4g n-3 PUFA, 1.9g EPA & DHA EPA/DHA:1.49/1;8-n-3 PUFA supplementation 3.5g/d;fish oil: 6 capsules/d (each capsule: 358mg EPA, 242mg DHA);flaxseed oil: 6 capsules/d (each capsule: 545mg w-3) | 1-Placebo(olive oil 4g/d);2-Replacement of 31g/1800kcal of dietary fat with 46g almond=31g oil;3-Placebo(paraffin);4-Placebo(olive oil 4g/d);5-Placebo(soybean oil 6 capsules/d) | total testosterone;SHBG levels | Yes(Random;Blinding;Incomplete;)No(Allocation;Selective;)Low |
| Sadeghi, A. | 2017 | Iran | 145 | 1-EPA/DHA dose 1.2 g;2-EPA/DHA dose 3.32 g;3-EPA/DHA dose 3.6 g | 1-Placebo;2-Placebo;3-Placebo | insulin resistance;HOMA –IR | Yes(Random;Blinding;Allocation;)No(Incomplete;Selective;)Low |
| Tosatti, Jessica A. G. | 2021 | Brazil | 384 | 1-2000 mg/d of fish oil per d;2-2000 mg/d of fish oil plus 50 000 IU of vitamin D every 2 weeks;3-180 mg of EPA and 120 mg of DHA per d;4-1000 mg/d of flaxseed oil;5-720 mg EPA and 480 mg DHA per d;6-540 mg EPA and 360 mg DHA per d;7-720 mg EPA and 480 mg DHA per d;8-400 mg of ALA plus 400 IU of vitamin E per d;9-400 mg of ALA plus 400 IU of vitamin E per d;10-545 mg ALA per d;11-358 mg EPA plus 242 mg DHA per d | 1-100 mg of paraffin oil per d;2-Not reported;3-1000 mg of paraffin oil per d;4-500 mg of paraffin oil per d;5-Four capsule contained 500 mg paraffin oil;6-1000 mg of paraffin oil per d;7-Paraffin oil;8-Not reported;9-Paraffin oil;10-Soya oil;11-Soya oil | adiponectin concentrations;visfatin concentrations;NO;GSH;MDA;TAC | Yes(Blinding;Incomplete;Selective;)No(Random;Allocation;)Low |
| Xia, Y. | 2021 | China | 778 | 1-[dosage (mg/d)] Fish oil (2,000);EPA (mg) 240;DHA (mg) 160;2-[dosage (mg/d)] Flaxseed oil (1,000) + VE (400 IU/d);EPA (mg) NA;DHA (mg) NA;3-[dosage (mg/d)] Fish oil (3,500);EPA (mg) 358;DHA (mg) 242;4-[dosage (mg/d)] Flaxseed oil (3,500);EPA (mg) 358;DHA (mg) 242;5-[dosage (mg/d)] Omega-3 supplement (2,000);EPA (mg) 180;DHA (mg) 120;6-[dosage (mg/d)] Omega-3 supplement (1,000);EPA (mg) 180;DHA (mg) 120;7-[dosage (mg/d)] Flaxseed oil (1,000);EPA (mg) NA;DHA (mg) NA;8-[dosage (mg/d)] Omega-3 supplement (4,000);EPA (mg) 180;DHA (mg) 120;9-[dosage (mg/d)] Omega-3 supplement (4,000);EPA (mg) 180;DHA (mg) 120;10-[dosage (mg/d)] Flaxseed oil (1,000) + VE (400 U/d);EPA (mg) NA;DHA (mg) NA;11-[dosage (mg/d)] Flaxseed oil (1,000) + VE (400 U/d);EPA (mg) NA;DHA (mg) NA | 1-Placebo;2-Placebo;3-Soybean oil;4-Soybean oil;5-Olive oil;6-Placebo;7-Placebo;8-Placebo;9-Placebo;10-Placebo;11-Placebo | insulin;HOMA-IR;total cholesterol (TC);triglyceride;LDL-C;VLDL-C;hs-CRP;HDL-C;serum glucose | Yes(Random;Blinding;Incomplete;Selective;)No(Allocation;)Moderate |
| Yang, K. | 2018 | China | 591 | 1-Omega-3 fatty acids 4000 mg;2-Omega-3 fatty acids (including fish oils and flaxseed oils);3-Omega-3 fatty acids 900 mg;4-Omega-3 fatty acids 900 mg;5-Omega-3 fatty acids 1000 mg + Vitamin E 400 IU + Metformin;6-Omega-3 fatty acids 1000 mg + vitamin E 400 IU;7-Omega-3 fatty acids 2000 mg;8-Omega-3 fatty acids 2000 mg + Metformin 500 mg;9-Omega-3 fatty acids 1000 mg + Vitamin E 400 IU | 1-Paraffin oil (placebo) 2000 mg;2-Soybean oil (placebo);3-Paraffin oil (placebo) 3000 mg;4-Paraffin oil (placebo) 3000 mg;5-Placebos + Metformin;6-Placebos;7-Olive oil (pla;8-cebo) 2000 mg;8-Paraffin oil (placebo) 1000 mg + Metformin 500 mg;9-Paraffin oil (placebo) | HOMA;total cholesterol;TG;Adiponectin;BMI;hyperinsulinemia;fasting glucose;LDL-C;HDL-C;FSH;LH;SHGB;total testosterone | Yes(Random;Allocation;Blinding;Incomplete;Selective;)High |
| Yuan, J. | 2021 | China | 610 | 1-Fish oil [2,000 mg/d];2-Flaxseed oil [1,000 mg/d] + VE (400 IU/d);3-Multinutrient [500 mg/d];4-Fish oil [2,000 mg/d] +VD (357 IU/d);5-Fish oil [3,500 mg/d];6-Flaxseed oil [3,500 mg/d];7-Flaxseed oil [1,000 mg/d];8-mega-3 supplement [3,000 mg/d];9-Flaxseed oil [1,000 mg/d] + VE (400 U/d);10-flaxseed oil [1,000 mg/d] + VE (400 IU/d);11-Fish oil [3,500 mg/d];12-Flaxseed oil [3,500 mg/d] | 1-Placebo;2-Placebo;3-Placebo;4-Placebo;5-Soybean oil;6-Soybean oil;7-Placebo;8-Placebo;9-Placebo;10-Placebo;11-Soybean oil;12-Soybean oil | CRP;GSH;MDA;TAC;DHEAS;FAI;FSH;LH;SHBG;TT | Yes(Random;Blinding;Incomplete;Selective;)No(Allocation;)Moderate |
| Zhou, J. | 2023 | China | 816 | 1-Fish oil n-3 PUFA sup- plements 2000 mg/d (Marine derived);2-Fish oil n-3 PUFA 4000 mg/d (Marine derived);3-Flaxseed oil n-3 PUFA 1000 mg/d (Plant origins)+vitamin E 400 IU/d;4-Fish oil n-3 PUFA 2000 mg/d (Marine derived)+vitamin E 50,000 IU/2 weeks;5-n-3 PUFA supple- ments 2000 mg/d (360 mg EPA and 240 mg DHA) (Marine derived);6-n-3 PUFA supple- ments 1000 mg/d (180 mg EPA and 120 mg DHA);7-Flaxseed oil n-3 PUFA 2000 mg/d (Plant origins);8-n-3 PUFA supple- ments 4000 mg/d (720 mg EPA and 480 mg DHA) (Marine derived);9-n-3 PUFA supple- ments 1000 mg/d (180 mg EPA and 120 mg DHA);10-Flaxseed oil n-3 PUFA 1000 mg/d (Plant origins)+Vita- min E 400 IU/d | 1-Parafn oil (placebo) 100 mg/d;2-olive oil (placebo) 4000 mg/d;3-Placebo;4-Placebo;5-Olive oil (placebo) 2000 mg/d;6-Parafn oil (placebo) 1000 mg/d;7-Parafn oil (placebo) 500 mg/d;8-Parafn oil (placebo) 500 mg/d;9-Parafn oil (placebo) 1000 mg/d;10-Placebo | BW;BMI;WC levels;FPG levels;FINS;HOMA-IR;QUICKI;Adiponectin;TG;TC;HDL-C;LDL-C;hs-CRP; | Yes(Random;Allocation;Blinding;Incomplete;Selective;)High |
| Abu-Zaid, A. | 2024 | Saudi Arabia | 345 | 1-Alpha-Lipoic Acid | 1-Placebo | BMI;FBS;HOMA-IR;insulin levels;estrogen;FSH;LH;testosterone | Yes(Random;Blinding;Selective;)No(Allocation;Incomplete;)Low |
| Fadlalmola, Hammad Ali | 2023 | Saudi Arabia | 218 | 1-Resveratrol 400 mg/twice daily, orally;2-Resveratrol 1500 mg/day, orally;3-Resveratrol 800 mg/day, orally;4-Resveratrol 1000  mg/day, orally | 1-placebo;2-placebo;3-placebo;4-placebo | Total testosterone;FSH;LH;Prolactin;TSH;DHEAS;pregnancy rates;Cholestero;HDL-C;LDL-C;Triglycerides;Acne score;C-reactive protein (CRP);Insulin;Sex hormone binding globulin (SHBG) | Yes(Random;Blinding;Incomplete;Selective;)No(Allocation;)Moderate |
| Abdelazeem, B. | 2022 | USA | 296 | 1-Highly bioavailable gel optimized Curcumin 93.34 mg QD;2-Curcumin 500 mg TID;3-Curcumin 500 mg QD;4-Curcumin 500 mg BID;5-Curcumin nanomicelle/ Metformin 80 mg QD/500 mg TID | 1-Placebo;2-Placebo (maltodextrin);3-Placebo (starch);4-Placebo;5-Metformin | fasting blood glucose;insulin level;homeostasis model assessment of insulin resistance;quantitative insulin sensitivity check index;total cholesterol; | Yes(Allocation;Blinding;Incomplete;Selective;)No(Random;)Moderate |
| Chien, Y. J. | 2021 | China Taiwan | 168 | 1-Curcumin 500 mg BID;2-Curcumin 500 mg TID;3-Curcumin 500 mg QD | 1-Placebo BID;2-Placebo(maltodextrin) TID;3-Placebo (starch) QD |  | Yes(Random;Blinding;Incomplete;)No(Allocation;Selective;)Low |
| Luis E. Simental‑Mendía | 2022 | Mexico | 296 | 1-Curcumin 1 g/day;2-Curcumin 93.34 mg/day;3-Curcumin 500 mg/day;4-Curcumin 1.5 g/day;5-Curcumin 80 mg/day+metformin 1.5 g/day | 1-Placebo;2-Placebo;3-Placebo;4-Placebo;5-Metformin 1.5 g/day | fasting glucose;insulin levels;HOMA-IR index;TC;LDL-C;HDL-C;triglycerides; | Yes(Random;Incomplete;Selective;)No(Allocation;Blinding;)Low |
| Mehran Nouri | 2022 | Iran | 198 | 1-curcumin 500(mg/d);2-curcumin 500(mg/d);3-curcumin 93.34(mg/d);4-curcumin 1500(mg/d) | 1-placebo;2-placebo;3-placebo;4-placebo | FBS;insulin level;HOMA-IR;QUICKY;BMI;insulin level; | No(Random;Allocation;Blinding;Incomplete;Selective;)Low |
| Shen, W. | 2022 | China | 447 | 1-Curcumin 500mg, qd;2-Curcumin 80mg, qd;Metformin 500mg, tid;3-Curcumin 500mg, bid;4-Curcumin 93.34mg;5-Curcumin 500mg, tid;6-CL water decoction 45ml, bid;7-CL water decoction 45ml, bid;Metformin 0.85g, bid | 1-Placebo Nr;2-Metformin 500mg, tid;3-Placebo Nr;4-Placebo Nr;5-Placebo 500mg, tid;6-Placebo 45ml,bid;7-Metformin 0.85g, bid | weight;waist circumference (WC);BMI;WHR;CRP;FBG;INS;QUICKI;HOMA-IR;2h OGTT;Ins120;glycosylated hemoglobin A1c (HbA1c);TC;TG;LDL-C;HDL-C;testosterone;dehydroepiandrosterone-sulfate (DHEA);LH;FSH;FAI;level of RBC;WBC;Cr;ALT;AST;adverse events; | Yes(Random;Incomplete;Selective;)No(Allocation;Blinding;)Low |
| Xie, Liangzhen | 2019 | China | 567 | 1-curcumin 500(mg/d);2-curcumin 500(mg/d);3-curcumin 93.34(mg/d);4-curcumin 1500(mg/d) | 1-placebo;2-placebo;3-placebo;4-placebo | pregnancy;total testosterone;SHBG;FAI;LH;fasting plasma glucose;insulin levels;postprandial plasma glucose level;HOMA-IR;total cholesterol;triglycerides;LDL-C;HDL-C;BMI;WC;WHR;gastrointestinal adverse events;events during pregnancy;live birth rate;conception;ovulation per subject;ovulation per cycle;Serious events during pregnancy | Yes(Random;)No(Allocation;Blinding;Incomplete;Selective;)Low |
| Caroline Cristine Almeida Balieiro | 2024 | Brazil | 916 | 1-Green tea;2-isoflavones(50 mg);3-Quercetin(1000 mg);4-Green tea;5-Quercetin(1000 mg);6-Quercetin(500 mg) | 1-Placebo;2-Placebo;3-Placebo;4-Placebo;5-Placebo;6-Placebo | insulin level;BMI;LH levels;testosterone levels; | Yes(Random;Blinding;Incomplete;Selective;)No(Allocation;)Moderate |
| Lesani, A. | 2022 | Iran | 139 | 1-540 mg/d Green tea extract capsule;2-500 mg/d Green tea capsule;3-500 mg/d Green tea tablet | 1-Placebo;2-Placebo;3-Placebo | weight;fasting insulin;waist to hip ratio;body mass index;body fat percentage | Yes(Random;Allocation;Blinding;Incomplete;Selective;)High |
| Heshmati, Javad | 2018 | Iran | 268 | 1-Chromium picolinate 200μg daily;2-Chromium picolinate 1000μg daily;3-Chromium picolinate 500mg twice daily;4-Chromium 200μg;5-Chromium picolinate 200μg | 1-Metformin;2-placebo;3-MET 500mg thrice daily;4-Placebo;5-Placebo | fasting insulin;QUICKI;HOMA-IR;FSI; | Yes(Random;Allocation;Blinding;Incomplete;Selective;)High |
| Siavash, Fazelian | 2017 | Iran | 351 | 1-200 mcg/day Cr picolinate;2-1000 mcg/day Cr picolinate;3-1000 mcg/day Cr picolinate;4-200 mcg/day Cr picolinate;5-200 mcg/day Cr picolinate;6-1000 mcg/day Cr picolinate;7-200 mcg/day Cr picolinate | 1-Placebo;2-nr;3-nr;4-Placebo(cellulose);5-Placebo(cellulose);6-placebo;7-1500 mg/day Metformin | FBS;fasting insulin;BMI;free testosterone;total testosterone;FG;DHEA;FSH;LH; | Yes(Random;Blinding;Incomplete;)No(Allocation;Selective;)Low |
| Ahmed Abu-Zaid | 2023 | Saudi Arabia | 413 | 1-Selenium dosage 200(μg/day);2-Selenium dosage 200(μg/day);3-Selenium dosage 200(μg/day);4-Selenium dosage 200(μg/day);5-Selenium dosage 200(μg/day);6-Selenium dosage 200(μg/day);7-Selenium dosage 200(μg/day) | 1-Placebo;2-Placebo;3-Placebo;4-Placebo;5-Placebo;6-Placebo;7-Placebo | FPG;insulin;HOMA-IR;QUICKI;TC;TG;HDL;LDL;VLDL;MDA;hs-CRP;TAC;GSH;NO;SHBG;total testosterone;MFG score;total testosterone; | Yes(Random;Allocation;Blinding;)No(Incomplete;Selective;)Low |
| Arentz, S. | 2017 | Australia | 1406 | 1-Selenium 200mcg per day;2-Selenium 200mcg plus Metformin 1500 mg per day; | 1-Placebo (type not specified);2-Placebo (cellulose) plus Metformin 1500 mg per day | fasting glucose;HOMA-IR;total testosterone;SHBG;FAI | Yes(Random;Allocation;)No(Blinding;Incomplete;Selective;)Low |
| Pei-yu Wu | 2022 | China | 317 | 1-200 μ g/day selenium for 12 weeks;2-200-μg selenium daily for 12 weeks;3-200 µg/day selenium for 12 weeks;4-200 µg/day selenium for 12 weeks;5-200 µg/day selenium for 8 weeks | 1-placebo;2-placebo;3-placebo;4-placebo;5-placebo | total testosterone;SHBG;cholesterol;triglyceride;LDL;FPG;HOMA-IR; | Yes(Random;Blinding;Incomplete;Selective;)No(Allocation;)Moderate |
| Ziaei, S. | 2024 | Iran | 1006 | 1-Melatonin dosage 6(mg/day);2-Melatonin dosage 10(mg/day);3-Melatonin dosage 10(mg/day);4-Melatonin dosage 6(mg/day);5-Melatonin dosage 3(mg/day);6-Melatonin dosage 3(mg/day) | 1-Placebo;2-Placebo;3-Placebo;4-Placebo;5-Placebo;6-Placebo | weight;BMI;TAC levels;FBS;Insulin;HOMA-IR;TC;TG;HDL;LDL;MDA;hs-CRP;mFG;SHGB;Total Testosterone;Endometrial thickness;Pregnancy Rate | Yes(Random;Allocation;Blinding;)No(Incomplete;Selective;)Low |
| Arentz, S. | 2017 | Australia | 1406 | 1-Inositol 2 g and folic acid 200 mg plus folic acid 200mcg;2-Inositol 4 g + folic acid 400mcg;3-Inositol 1200 mg/day as powder,pre-dosed presented in sachets dissolved in water;4-Inositol 200 mg;5-Inositol 4 g plus folic acid 400mcg;6-Inositol (chiro) 600 mg;7-Inositol 4 g + folic acid 400mcg | 1-Folic acid 400mcg daily;2-Folic acid 400mcg;3-Placebo;4-Placebo matched to Gestosan;5-Folic acid 400mcg;6-Placebo;7-Metformin | Free testosterone;Total testosterone;Androstenidione;Sex hormone binding globulin (SHBG);Modified Ferriman Gallwey score;Number of days to ovulation;Number of ovulations;Pregnancy;Live births;FSH;LH;FSH:LH ratio;BMI;Waist to hip ratio;Fasting glucose;Fasting insulin;HOMA-IR;Cholesterol;Triglycerides; | Yes(Random;Allocation;)No(Blinding;Incomplete;Selective;)Low |
| Greff, D. | 2023 | Hungary | 1691 | 1-MI 1000 mg 24w;2-MI (1100 mg)+DCI (27,6 mg) 24w;3-MI (2000 mg)+DCI (400 mg) 12w;4-MI 1000 mg 16w;5-MI 4000 mg 12w;6-MI 4000 mg 12-16w;7-MI 4000 mg 12-16w;8-1. MI 4000 mg 24w;2. MI 1100 mg+DCI 27,6 mg 24w;9-MI 4000 mg 24w;10-MI 2000 mg 12w;11-MI 4000 mg 14w;12-MI 4000 mg 12w;13-DCI 600 mg 7w;14-MI 3000 mg 24w;15-MI 4000 mg 12w;16-MI 2000 mg 24w;17-MI 2000 mg 24w;18-DCI 1200 mg 7w;19-MI 4000 mg 12w;20-MI 4000 mg 24w;21-MI 4000 mg 12w;22-MI 4000 mg NR w;23-MI 4000 mg 12w;24-MI 4000 mg 12w;25-MI 4000 mg 24w;26-MI 1000 mg 24w | 1-MET 1000 mg;2-FA 400mcg;3-FA 400mcg;4-MET 1000 mg;5-MET 1700 mg;6-FA 400 mcg;7-Placebo powder;8-diet;9-MET 1500 mg;10-FA 200mcg;11-FA 400mcg;12-MET 1500 mg;13-NR;14-MET 1700 mg;15-MET 1500 mg;16-MET 1500 mg;17-MET 1500 mg;18-Placebo;19-1.MET 1500 mg;2.FA 400mcg;20-MET 1500 mg;21-MET 1700 mg;22-FA 400mcg;23-MET 1500 mg;24-FA 500mcg;25-MET 1500 mg;26-MET 1700 mg | the rate of cycle normalization;BMI;weight;total testosterone levels;free testosterone;SHBG levels;Androstenedione;androstenedione;DHEAS;fasting plasma glucose;glucose levels;AUC-insulin levels;pregnancy rate;cycle normalization;SHBG;FG-score;pregnancy rate;Side efects; | Yes(Random;Blinding;)No(Allocation;Incomplete;Selective;)Low |
| Pundir, J. | 2017 | UK | 601 | 1-Inositol (Myo-inositol or di-chiro-inositol);2-Myo-inositol;3-Myo-inositol;4-Myo-inositol;5-Inositol (Myo-inositol or di-chiro-inositol) | 1-placebo;2-Di-chiro-inositolvs. placebo;3-Di-chiro-inositol;4-metformin;5-Clomiphene | ovulation rate;frequency of menstrual cycles;pregnancy;total androgen;total testosterone;free testosterone;serum dehydroepiandrosterone;sex-hormone-binding globulin;fasting insulin;fasting glucose;HOMA-IR; | Yes(Random;Blinding;Incomplete;Selective;)No(Allocation;)Moderate |
| Unfer, Vittorio | 2017 | Switzerland | 486 | 1-MI+FA;2-MI+FA;3-MI+FA;4-MI+FA;5-MI+FA;6-MI+FA;7-MI+FA;8-MI+DCI;9-MI+DCI | 1-FA;2-FA;3-FA;4-FA;5-DCI+FA;6-OCPs;7-COC;8-MI;9-FA | fasting insulin;HOMA-IR;serum testosterone;androstenedione;SHBG levels; | Yes(Incomplete;Selective;)No(Random;Allocation;Blinding;)Low |
| Zeng, L. | 2018 | China | 573 | 1-MI 0.55 g, DCI 0.0138 g and folic acid 200 μg b.i.d;2-MI 1 g b.i.d;3-MI 4 g and folic acid 400 μg q.d.;4-MI 2 g and folic acid 200 μg q.d.;5-MI 1.2 g q.d.;6-MI 4 g and folic acid 400 μg q.d.;7-MI 2 g and folic acid 200 μg q.d.;8-Group I: MI 2 g and folic acid 200 μg; Group III MI 2 g, folic acid 200 μg and drospirenone 3 mg/ethinyl estradiol 30 μg;9-MI 2 g and folic acid 200 μg b.i.d;10-MI 0.1 g b.i.d | 1-Folic acid 200 μg b.i.d;2-Metformin 500 mg t.i.d.;3-Metformin 1.5 g q.d.;4-Folic acid 400 μg q.d.;5-Placebo 1.2 g q.d.;6-Folic acid 400 μg q.d.;7-Folic acid 200 μg q.d.;8-Group II:Drospirenone 3 mg/ethinyl estradiol 30μg;9-Folic acid 200 μg b.i.d;10-Placebo 0.1 mg b.i.d | HOMA-IR;total testosterone;fasting glucose;free testosterone;SHBG;androstenedione;FSH;LH; DHEAS | Yes(Random;Blinding;Incomplete;)No(Allocation;Selective;)Low |
| Hajar Heidari | 2022 | Iran | 504 | 1-1000 mg omega-3 fatty acids plus 400 IU vitamin E supplements；2-1000 mg omega-3 fatty acids from faxseed oil  containing 400 mg α-linolenic acid plus 400 IU vitamin E supplements；3-1000 mg omega-3 fatty acids from faxseed oil containing 400 mg α-Linolenic acid plus 400 IU vitamin E supplements；4-400 IU vitamin E；5-250 mg/day magnesium plus 400 mg/ day vitamin E supplements；6-400 IU vitamin E；7-1000 mg omega-3 plus 400 IU vitamin E supplements；8-250 mg/day magnesium plus 400 mg/day vitamin E supplements；9-2 g of omega-3 plus 400 IU of vitamin E；10-400 IU/day vitamin E -as alpha tocopheryl acetate | 1-Placebo (parafn)；2-Placebo；3-Placebo；4-Placebo；5-Placebo；6-Placebo；7-Placebo；8-Placebo；9-Placebo；10-Placebo | TG;VLDL;LDL-c;TC;TC /HDL-c ratio;TAC;GSH;MDA;hs-CRP;NO levels;FBS;insulin;HOMA-IR;QUICKI;total testosterone;LH;FSH;SHBG;FAI;weight;BMI;waist circumference;hirsutism score | Yes(Random;Blinding;Incomplete;Selective;)No(Allocation;)Moderate |
| Sebastián Yalle-Vásquez | 2022 | Peru | 540 | 1-Vitamin E 400 IU þ Omega-3 1000 mg daily for 3 months;2-Vitamin E 400 mg þ 50 Vitamin D3000 IU/L twice a week or 3 300 IU/day for 10 weeks;3-Vitamin E 400 IU daily for 2 months;4-Vitamin E 400 IU þ Omega-3 100 mg daily for 3 months;5-Vitamin E 400 mg þ Magnesium oxide 250 mg daily for 3 months;6-Vitamin E 1500 IU daily for 3 menstrual cycles;7-Vitamin E 300 mg/dl 3 times daily for 2 months;8-Vitamin E 400 IU daily for 2 months | 1-placebo;2-placebo;3-placebo;4-placebo;5-placebo;6-placebo;7-placebo;8-placebo | fasting serum glucose;HOMA-IR;fasting serum insulin;BMI;testosterone;free androgen index;SHBG;Ferriman-Gallwey score;total cholesterol;LDL-cholesterol;triglyceride levels;HDL-cholesterol levels | Yes(Random;Incomplete;Selective;)No(Allocation;Blinding;)Low |
| Akbari, M. | 2018 | Iran | 406 | 1-1000 IU vitamin D/day;2-50000 IU vitamin D/every 2 weeks;3-1000 IU vitamin D/day + 1 g EPO/day;4-400 IU vitamin D/day + other nutrients/day;5-400 IU Vitamin D/day + 180 μg vitamin K/day + 1 g calcium carbonate/day;6-50000 IU vitamin D/week + 1 g calcium carbonate/day;7-50000 IU vitamin D/every 20 days | 1-placebo;2-placebo;3-placebo;4-placebo;5-placebo;6-placebo;7-placebo | hs-CRP;NO;TAC levels;GSH;MDA | Yes(Random;Allocation;Blinding;Selective;)No(Incomplete;)Moderate |
| Fang, F. 2017 | 2017 | China | 502 | 1-Vitamin D 50000 IU/20 days;2-Vitamin D 50000 IU/20 days;3-Vitamin D 50000 IU/week + Ca 1000 mg/day;Vitamin D 50000 IU/week + Ca placebo;4-Vitamin D 0.5 mg/day;5-Vitamin D 50000 IU/week + Ca 1000 mg/day + merformin 1500 mg/day;6-Vitamin D 4000 IU/day + metformin 1500 mg/day;7-Vitamin D 12000 IU/day;8-Vitamin D 400 IU/day + Ca 1000 mg/day + metformin 1500 mg/day;Vitamin D 400 IU/day + Ca 1000 mg/day;9-Vitamin D 50,000 IU/2 weeks + Ca 1000 mg/day + metformin 1500 mg/day;Vitamin D 50000 IU/2 weeks + Ca 1000 mg/day | 1-Placebo;2-Placebo;3-Vitamin D placebo + Ca 1000 mg/day;Vitamin D placebo + Ca placebo;4-Metformin 1000 mg/day;Placebo;5-Metformin 1500 mg/day;6-Metformin 1500 mg/day + placebo;Placebo;7-Metformin 1500 mg/day;8-Metformin 1500 mg/day;9-Placebo | serum 25(OH)D;Serum PTH levels;the number of dominant follicles;regular menstrual cycles;follicle development | Yes(Random;Allocation;Blinding;Incomplete;Selective;)High |
| Gao, H. | 2021 | China | 543 | 1-Vitamin D | 1-Placebo | total cholesterol level;triglyceride level;LDL-C; | Yes(Random;Allocation;Selective;)No(Blinding;Incomplete;)Low |
| Han, Y. | 2023 | China | 849 | 1-Vitamin D + calcium;2-Vitamin D;3-Vitamin D + calcium + vitamin K2;4-Metformin + vitamin D;5-Metformin + calcium + vitamin D;6-Vitamin D;7-Vitamin D;vitamin D + metformin;8-Metformin + calcium + vitamin D;9-Vitamin D;10-Vitamin D + calcium;Metformin + calcium + vitamin D;11-Metformin + calcium + vitamin D;Vitamin D + calcium;12-Vitamin D | 1-Calcium;2-Placebo;3-Placebo;4-Metformin;5-Metformin;6-Placebo;7-Metform;8-Metformin;9-Placebo;10-Metformin;11-Placebo;Metformin;12-Placebo | LH;FSH;LH/FSH;menstrual cycle; | Yes(Random;Incomplete;Selective;)No(Allocation;Blinding;)Low |
| Jin, B. | 2020 | China | 467 | 1-Cholecalciferol;2-Cholecalciferol;3-Cholecalciferol;4-Cholecalciferol + Calcium;5-Cholecalciferol + MET;6-Cholecalciferol;7-Cholecalciferol(high-dose) + MET;Cholecalciferol(lose-dose) + MET;8-Cholecalciferol;9-Cholecalciferol | 1-Placebo;2-Placebo;3-Placebo;4-Placebo + Calcium;5-Placebo + MET;6-Placebo;7-Placebo + MET;8-Placebo;9-Placebo | triglyceride levels;total cholesterol;low-density lipoprotein cholesterol;very-low-density lipoprotein cholesterol;high-density lipoprotein cholesterol levels | Yes(Random;Blinding;Incomplete;Selective;)No(Allocation;)Moderate |
| Luo, J. | 2021 | China | 677 | 1-Vitamin D;2-Vitamin D;Vitamin D + calcium;3-Vitamin D;4-Low-dose vitamin D;High-dose vitamin D;5-Vitamin D + metformin;6-Vitamin D;7-Vitamins D and K + calcium;8-Vitamin D;9-Vitamin D + EPO;10-Vitamin D;11-Vitamin D | 1-Placebo;2-Placebo;3-Placebo;4-Placebo;5-Placebo + metformin;6-Placebo;7-Placebo;8-Placebo;9-Placebo;10-Placebo;11-Placebo | TC;TG;LDL-C;HDL;VLDL-C | Yes(Random;Allocation;Blinding;Selective;)No(Incomplete;)Moderate |
| Miao, C. Y. | 2019 | China | 483 | 1-Weight-loss intervention + 50,000 IU/week oral vitamin D3;2-50,000 IU of oral vitamin D3;3-50,000 IU vitamin D every 2 weeks;4-50,000 IU of vitamin D3 every 20 days;5-Vitamin D3 12,000 IU daily;6-Metformin (1,500 mg/day) + vitamin D 4,000 IU/day (monthly dose of 120,000 IU);7-50,000 IU vitamin D every other week;8-Vitamin D 3200 IU daily;9-50,000 IU of oral vitamin D3 once weekly;10-50,000 IU vitamin D weekly and calcium placebo daily;11-Calcitriol 0.5 µg/day | 1-Weight loss intervention + placebo;2-Placebo;3-Placebo;4-Placebo;5-Placebo;6-Metformin (1,500 mg/day) + placebo;7-Placebo;8-Placebo;9-Placebo once weekly;10-Calcium placebo daily plus vitamin D placebo weekly;11-Placebo | BMI;total testosterone;DHEA;HOMA-IR;total cholesterol;LDL-C;triglyceride;HDL-C | Yes(Random;Allocation;)No(Blinding;Incomplete;Selective;)Low |
| Pergialiotis, V. | 2017 | Greece | 647 | 1-Ca+vD;Ca+vD+Met;2-Met;vD;3-Vitamin D;4-Met;Met+Ca+vD;Ca+vD;5-vD3;6-vD+Met;7-vD;8-vD+vK+Ca;9-vD;vD+Met | 1-Met;2-placebo;3-placebo;4-placebo;5-placebo;6-placebo;7-placebo;8-placebo;9-Met | testosterone;LH;DHEAS;fasting glucose;fasting insulin;HOMA-IR; | Yes(Random;Allocation;Incomplete;Selective;)No(Blinding;)Moderate |
| Xue, Y. | 2017 | China | 855 | 1-nr;2-Vitamin D: 50,000 IU/wk 8 weeks;3-Vitamin D3: 50,000 IU/20 days 2 months;4-Vitamin D:400 IU orally 3 months;5-Vitamin D3:10,0000 IU/month 6 months;6-Vitamin D: 3,300,000 IU/day as a single oral dose 3 weeks;7-Cholecalciferol:300,000 IU,by intragluteal injection;8-Vitamin D3:4000 IU/day 6 months;9-Calcitriol:0.5 lg/day 3 month;10-Vitamin D3: 2000 IU/day + Vitamin D2 50,000 IU/month;11-Vitamin D3: 12000 IU/12 weeks 12 weeks;12-Vitamin D3: 20,000 IU/week 12 weeks;13-Vitamin D3: 50,000 IU/ 2 weeks 4 months;14-Ergocalciferol 50 000 IU weekly or biweekly;15-Vitamin D3: 50 000 IU oral 8 weeks;16-Vitamin D: 200 IU 8 weeks | 1-Placebo;2-Placebo;3-Placebo;4-Placebo;5-Placebo;6-Placebo;7-Placebo;8-Placebo;9-Placebo;10-Placebo;11-Placebo;12-Placebo;13-Placebo;14-Placebo;15-Placebo;16-Placebo | 25-hydroxy-vitamin D;HOMA-IR;QUICKI;serum triglyceride;LDL;PTH;DHEAS;FT;TT | Yes(Random;Allocation;Blinding;Incomplete;Selective;)High |
| Zhang, B. | 2023 | China | 840 | 1-VD3 300,000 IU;2-VD 4000 IU/day;3-VD 50,000 IU/week;4-VD 4000 or 1 000IU/day;5-VD 50,000 IU/2 weeks;6-VD 50,000 IU/2 weeks;7-200 IU VD/day;8-VD 50,000 IU/2 weeks;9-VD3 50,000 IU;10-VD3 12,000 IU/day;11-VD 200 IU/day;12-VD 200 IU/day;13-VD 400 IU/day | 1-Placebo;2-Placebo;3-Placebo;4-Placebo;5-Placebo;6-Placebo;7-Placebo;8-Placebo;9-Placebo;10-Placebo;11-Placebo;12-Placebo;13-Placebo | hs-CRP;Parathyroid hormone level;Total cholesterol level;Total testosterone level;(mF-G) score;Endometrial thickness | Yes(Random;Allocation;Incomplete;Selective;)No(Blinding;)Moderate |
| Li, Y. | 2023 | China | 1049 | 1-Probiotic;2-Probiotic;3-Synbiotic;4-Probiotic;5-Probiotic;6-Probiotic;7-Synbiotic;8-Synbiotic;9-Synbiotic;10-Probiotic;11-Synbiotic;12-Prebiotic;13-Probiotic;14-Prebiotic;15-Probiotic;16-Synbiotic;17-Probiotic | 1-Placebo (starch);2-Placebo;3-Placebo;4-Placebo(maltodextrin);5-Placebo;6-Placebo (starch);7-Placebo (starch and maltodextrin);8--Placebo (starch and maltodextrin);9-Placebo;10-Placebo (starch);11-Placebo (starch);12-Placebo (maltodextrin);13-Placebo;14-Placebo(maltodextrin);15-Placebo (starch and maltodextrin);16-Placebo;17-Placebo | FPG;FINS;HOMA-IR;QUICKI;TG;TC;HDL-c;LDL-c;VLDL-c;BW;BMI;WC;HC;CRP; | Yes(Random;Allocation;Incomplete;Selective;)No(Blinding;)Moderate |
| Miao, C. | 2021 | China | 486 | 1-Lactobacillus rhamnosus GG, Bacillus coagulans, Bacillus indicus;2-Lactobacillus acidophilus, Lactobacillus casei, Lactobacillus bulgaricus, Lactobacillus rham- nosus, Bifidobacterium longus, Bifidobacterium breve, Streptococcus thermophilus;3-Lactobacillus acidophilus, Lactobacillus casei, Bifidobacterium bifidum;4-Lactobacillus casei, Lactobacillus acidophilus, Lactobacillus rham- nosus Lactobacillus, Bulgaricus, Bifidobacterium breve, Bifidobacterium longum, Streptococcus thermophiles;5-20 g of prebiotic;6-Lactobacillus acidophilus, Lactobacillus casei, Lactobacillus bulgaricus, Lactobacillus rham- nosus, Bifidobacterium longum, Bifidobacterium breve, Streptococcus thermophilus;7-Bifid triple viable combined with metformin | 1-Unknown;2-Placebo capsules containing starch and maltodextrin, but no bacteria;3-Placebo;4-Placebo containing starch and maltodextrins but no bacteria;5-Placebo (maltodextrin);6-Placebo capsules containing starch and maltodextrin but no bacteria;7-Metformin | HOMA-IR;insulin;FBS;BMI;WC;HC | Yes(Random;Allocation;Incomplete;Selective;)No(Blinding;)Moderate |
| Reza, Tabrizi | 2022 | Iran | 730 | 1-Synbiotic capsule contained Lactobacillus acidophilus, Lactobacillus casei, and Bifidobacterium bifidum (2 × 109 CFU/g each) plus 800 mg inulin;2-Synbiotic capsule (500 mg) contained Lactobacillus acidophilus 3 × 1010 CFU/g, Lactobacillus casei 3 × 109 CFU/g, Lactobacillus bulgaricus 5 × 108 CFU/g, Lactobacillus rhamnosus 7 × 109 CFU/g, Bifidobacterium longum 1 ×109 CFU/g,Bifidobacterium breve 2 × 1010 CFU/g and Streptococcus thermophilus 3 × 108 CFU/g + prebiotic inulin(fructo-oligosaccharide);3-Probiotic capsule contained Lactobacillus acidophilus, Lactobacillus casei and Bifidobacterium bifidum (2 × 109 CFU/g each);4-Probiotic capsule (500 mg) contained Lactobacillus casei 7 × 109 CFU/g, Lactobacillus acidophilus 2 × 109 CFU/g,Lactobacillus rhamnosus 1.5 × 109 CFU/g, Lactobacillus bulgaricus 2 × 108 CFU/g, Bifidobacterium breve 2 × 1010 CFU/g,Bifidobacterium longum 7 × 109 CFU/g, Streptococcus thermophiles 1.5 × 109 CFU/g;5-Probiotic capsule contained Lactobacillus acidophilus, Lactobacillus casei and Bifidobacterium bifidum (2 × 109 CFU/g each);6-Probiotic capsule (1000 mg) contained Lactobacillus acidophilus,Lactobacillus plantarum, Lactobacillus fermentum, and Lactobacillus Gasseri (1 × 109 CFU of each);7-Synbiotic capsule contained Lactobacillus acidophilus, Lactobacillus casei, and Bifidobacterium bifidum (2 × 109 CFU/g each) plus 800 mg inulin;8-Probiotic capsule contained Lactobacillus acidophilus, Lactobacillus reuteri, Lactobacillus fermentum, and Bifidobacterium bifidum(2 × 109 CFU/g each) + 200 mcg selenium;9-Probiotic capsule contained Lactobacillus acidophilus, Lactobacillus reuteri, Lactobacillus fermentum, and Bifidobacterium bifidum(2 × 109 CFU/g each) + 200 mcg selenium;10-Synbiotic pomegranate juice 2 lit per week contained Lactobacillus(4 × 108 CFU/g) plus 40 g inulin;11-Synbiotic beverage 2 lit per week contained Lactobacillus(4 × 108 CFU/g) plus 40 g inulin;12-Probiotic capsule contained Lactobacillus acidophilus, Lactobacillus reuteri, Lactobacillus fermentum, and Bifidobacterium bifidum(2 × 109 CFU/g each) + 50,000 IU vitamin D every 2 weeks | 1-Placebo;2-Placebo;3-Placebo;4-Placebo;5-Placebo;6-Placebo;7-Placebo;8-Placebo;9-Placebo;10-Placebo;11-Placebo;12-Placebo | weight;BMI;FPG;insulin;HOMA-IR;triglycerides;VLDL-cholesterol;CRP;malondialdehyde (MDA);hirsutism;total testosterone levels;QUICKI;NO;total antioxidant capacity (TAC);glutathione (GSH);sex hormone binding globulin (SHBG) | Yes(Random;Allocation;Blinding;Selective;)No(Incomplete;)Moderate |
| Shamasbi, S. G. | 2020 | Iran | 855 | 1-receive either one synbiotics capsule per day for 12 weeks;2-intake either probiotic supplements for 12 weeks;3-receive either probiotic supplements for 12 weeks;4-two probiotic capsules per day for 12 weeks;5-probiotic supplement;probiotic group received one Familact probiotic capsule (500 mg);6-Participants in the intervention group received ten billions probiotic capsules twice daily, after lunch and evening meal for 12 weeks;7-200 µg/day selenium as selenium yeast plus 8000000000 CFU/day probiotic;8-active treatment with synbiotics;9-Prebiotic containing 20 g of resistant dextrin (polysaccharides produced  from maize, wheat and other edible starches);10-Synbiotics supplements were containing Lactobacillus acidophilus, Lactobacillus casei and Bifdobacterium bifdum plus 0.8 g inulin;11-intake either probiotic plus 200 μg/day selenium;12-1. Synbiotics pomegranate juice;2. Pomegranate juice;3. Synbiotics beverage;13-Prebiotic containing 20 g of resistant dextrin (polysaccharides produced from maize, wheat and other edible starches) | 1-placebo;2-placebo;3-placebo (starch);4-two maltodextrin capsules;5-placebo;6-placebo;7-placebo;8-placebo;9-placebo containing 20 g maltodextrin;10-placebo;11-placebo;12-2 L(2×1-l) of placebo beverage per 1 week, for 8 weeks in disposable bottles (each liter of beverage contains 1 l of water+2 pomegranate favoring);13-placebo containing 20 g maltodextrin | Testosterone;DHEAS;SHBG;FAI;hsCRP;NO;TAC;GSH;MDA;hirsutism | Yes(Allocation;)No(Random;Blinding;Incomplete;Selective;)Low |
| Tabrizi, R. | 2022 | Iran | 730 | 1-Synbiotic capsule contained Lactobacillus acidophilus, Lactobacillus casei, and Bifidobacterium bifidum plus 800 mg inulin;2-Synbiotic capsule (500 mg) contained Lactobacillus acidophilus, Lactobacillus casei, Lactobacillus bulgaricus, Lactobacillus rhamnosus, Bifidobacterium longum,Bifidobacterium breve,Streptococcus thermophilus+ prebiotic inulin(fructo-oligosaccharide);3-Probiotic capsule contained Lactobacillus acidophilus, Lactobacillus casei and Bifidobacterium bifidum;4-Probiotic capsule (500 mg) contained Lactobacillus casei, Lactobacillus acidophilus,Lactobacillus rhamnosus,Lactobacillus bulgaricus,Bifidobacterium breve,Bifidobacterium longum,Streptococcus thermophiles;5-Probiotic capsule contained Lactobacillus acidophilus, Lactobacillus casei and Bifidobacterium bifidum;6-Probiotic capsule (1000 mg) contained Lactobacillus acidophilus, Lactobacillus plantarum, Lactobacillus fermentum, and Lactobacillus Gasseri;7-Synbiotic capsule contained Lactobacillus acidophilus, Lactobacillus casei, and Bifidobacterium bifidum plus 800 mg inulin;8-Probiotic capsule contained Lactobacillus acidophilus, Lactobacillus reuteri, Lactobacillus fermentum, and Bifidobacterium bifidum + 200 mcg selenium;9-Probiotic capsule contained Lactobacillus acidophilus, Lactobacillus reuteri, Lactobacillus fermentum, and Bifidobacterium bifidum + 200 mcg selenium;10-Synbiotic pomegranate juice 2 lit per week contained Lactobacillus plus 40 g inulin;11-Synbiotic beverage 2 lit per week contained Lactobacillus plus 40 g inulin;12-Probiotic capsule contained Lactobacillus acidophilus, Lactobacillus reuteri, Lactobacillus fermentum, and Bifidobacterium bifidum + 50,000 IU vitamin D every 2 weeks | 1-placebo;2-placebo;3-placebo;4-placebo;5-placebo;6-placebo;7-placebo;8-placebo;9-placebo;10-placebo;11-placebo;12-placebo | weight;BMI;fasting plasma glucose (FPG);insulin;HOMA-IR;triglycerides;VLDL-cholesterol;CRP;malondialdehyde (MDA);hirsutism;total testosterone levels;QUICKI;NO;total antioxidant capacity (TAC);glutathione (GSH);sex hormone binding globulin (SHBG) levels | Yes(Random;Allocation;Blinding;Selective;)No(Incomplete;)Moderate |
| Gong, Y. | 2023 | China | 839 | 1-Metformin tablets at the initial dose of 500 mg and gradually increased to not more than 1500 mg daily;Carnitine, 250 mg daily;2-Carnitine, 250 mg daily;3-150 mg/day CC for 5 days plus oral and metformin 850 mg twice daily;L‐carnitine, 3000 mg daily;4-Metformin tablets at the initial dose of 500 mg and gradually increased up to 1500 mg daily;Carnitine, 250 mg daily;5-Combined metformin and pioglitazone (500/15 twice daily);Carnitine, 3000 mg daily;6-CC (250 mg per day) from Day 3 to 7 days of the cycle;Carnitine, 3000 mg daily;7-CC;Carnitine, 1000 mg daily | 1-Metformin tablets at the initial dose of 500 mg and gradually increased to not more than 1500 mg daily;Placebo;2-Placebo;3-150 mg/day CC for 5 days plus oral and metformin 850 mg twice daily;Placebo;4-Metformin tablets at the initial dose of 500 mg and gradually increased up to 1500 mg daily;Placebo;5-Combined metformin with pioglitazone (500/15 twice daily);Placebo;6-CC (250 mg per day) from Day 3 to 7 days of the cycle;Placebo;7-CC;Placebo | ovulation rates;pregnancy rates;insulin;HOMA-IR;BMI; | Yes(Random;Blinding;Selective;)No(Allocation;Incomplete;)Low |
| Shukri, Mohd Falihin Mohd | 2022 | Malaysia | 995 | 1-150 mg/day CC plus oral LC 3g and metformin 850 mg (1 tablet daily);2-150 mg/day of CC plus 3 g of oral LC daily, and placebo sachets;3-250 mg CC from day three until day seven of the cycle plus LC 3 g daily;4-250 mg carnitine supplements;5-LC 1,000 mg/d plus 200 mg/d chromium as chromium picolinate;6-200 µg/day chromium pi- colinate plus 1,000 mg/day LC;7-250 mg LC (capsule range 237–275 mg);8-250 mg/day of LC;9-Oral CC (50 mg tablet, two times per day) plus oral LC supplementation (1 g tablet, three times per day) | 1-150 mg/d CC plus metformin and placebo capsules;2-150 mg/day of CC from day 3 until day 7 of the menstrual cycle plus 600 mg of oral N-acetylcysteine three times daily, and a placebo capsule;3-250 mg CC with placebo;4-Placebos (cellulose);5-Placebo;6-Placebo (starch);7-Placebo (cellulose);8-Placebo;9-Oral CC only (50 mg tablet, two times per day) | fasting plasma glucose (FPG);LDL;total cholesterol;triglyceride(TG); | Yes(Random;Allocation;Blinding;Incomplete;Selective;)High |

**Note**: EPA - Eicosapentaenoic Acid; DHA - Docosahexaenoic Acid; PUFA - Polyunsaturated Fatty Acids; SFA - Saturated Fatty Acids; MUFA - Monounsaturated Fatty Acids; LA - Linoleic Acid; ALA - Alpha-Linolenic Acid; HOMA-IR - Homeostasis Model Assessment of Insulin Resistance; LDL - Low-Density Lipoprotein; TC - Total Cholesterol; VLDL - Very Low-Density Lipoprotein; HDL-C - High-Density Lipoprotein Cholesterol; hs-CRP - High-Sensitivity C-Reactive Protein; NO - Nitric Oxide; GSH – Glutathione; MDA – Malondialdehyde; TAC - Total Antioxidant Capacity; SHBG - Sex Hormone Binding Globulin; FSH - Follicle Stimulating Hormone; LH - Luteinizing Hormone; DHEAS - Dehydroepiandrosterone Sulfate;FAI - Free Androgen Index; BMI - Body Mass Index; FBG - Fasting Blood Glucose; INS – Insulin; QUICKI - Quantitative Insulin Sensitivity Check Index; FSI - Follicle Stimulating Hormone Index; FG - Follicular Growth; mFG - Modified Ferriman Gallwey score; PCOS - Polycystic Ovary Syndrome; EPA - Eicosapentaenoic Acid; DHA - Docosahexaenoic Acid; w-3 - Omega-3; EPA - Eicosapentaenoic Acid; DHA - Docosahexaenoic Acid; ALA - Alpha-Linolenic Acid; TID - Three times a day; BID - Two times a day; QD - Once a day; VD - Vitamin D; vK - Vitamin K; Ca – Calcium; EPO – Erythropoietin; PTH - Parathyroid hormone; AUC - Area Under the Curve; DHEAS - Dehydroepiandrosterone Sulfate; FA - Folic Acid; DCI - D-Chiro-Inositol; MI - Myo-Inositol; CC - Clomiphene Citrate; MET – Metformin; FAI - Free Androgen Index; Cr – Chromium; Cr picolinate - Chromium Picolinate; CFU - Colony Forming Units; IU - International Units; VD3 - Vitamin D3; FG - Ferriman Gallwey score; mF-G - Modified Ferriman Gallwey score; TAC - Total Antioxidant Capacity; NO - Nitric Oxide; GSH – Glutathione; MDA – Malondialdehyde; CRP - C-Reactive Protein; FPG - Fasting Plasma Glucose; FINS - Fasting Insulin; TG – Triglycerides; TC - Total Cholesterol; HDL - High-Density Lipoprotein; LDL - Low-Density Lipoprotein; VLDL - Very Low-Density Lipoprotein; BW - Body Weight; WC - Waist Circumference; HC - Hip Circumference; CRP - C-Reactive Protein; RBC - Red Blood Cells; WBC - White Blood Cells; AST - Aspartate Aminotransferase; ALT - Alanine Aminotransferase; DHEA – Dehydroepiandrosterone; FAI - Free Androgen Index; TID - Three times daily; BID - Twice daily; QD - Once daily; VD - Vitamin D; NR - Not reported; CL - Not specified; FA - Folic Acid; DCI - D-Chiro-Inositol; MI - Myo-Inositol; CC - Clomiphene Citrate; MET – Metformin; TAC - Total Antioxidant Capacity; NO - Nitric Oxide; GSH – Glutathione; MDA – Malondialdehyde; CRP - C-Reactive Protein; FPG - Fasting Plasma Glucose; FINS - Fasting Insulin; HOMA-IR - Homeostasis Model Assessment of Insulin Resistance; QUICKI - Quantitative Insulin Sensitivity Check Index; TG – Triglycerides; TC - Total Cholesterol; HDL-C - High-Density Lipoprotein Cholesterol; LDL-C - Low-Density Lipoprotein Cholesterol; VLDL-C - Very Low-Density Lipoprotein Cholesterol; BW - Body Weight; BMI - Body Mass Index; WC - Waist Circumference; HC - Hip Circumference; CRP - C-Reactive Protein
